# Epigenome-Wide Meta-Analysis of Methylation in Children Related to Prenatal NO_2_ Air Pollution Exposure

**DOI:** 10.1289/EHP36

**Published:** 2016-07-22

**Authors:** Olena Gruzieva, Cheng-Jian Xu, Carrie V. Breton, Isabella Annesi-Maesano, Josep M. Antó, Charles Auffray, Stéphane Ballereau, Tom Bellander, Jean Bousquet, Mariona Bustamante, Marie-Aline Charles, Yvonne de Kluizenaar, Herman T. den Dekker, Liesbeth Duijts, Janine F. Felix, Ulrike Gehring, Mònica Guxens, Vincent V.W. Jaddoe, Soesma A. Jankipersadsing, Simon Kebede Merid, Juha Kere, Ashish Kumar, Nathanael Lemonnier, Johanna Lepeule, Wenche Nystad, Christian Magnus Page, Sviatlana Panasevich, Dirkje Postma, Rémy Slama, Jordi Sunyer, Cilla Söderhäll, Jin Yao, Stephanie J. London, Göran Pershagen, Gerard H. Koppelman, Erik Melén

**Affiliations:** 1 Institute of Environmental Medicine, Karolinska Institutet, Stockholm, Sweden; 2 Groningen Research Institute for Asthma and COPD (GRIAC), Department of Pulmonology, and; 3 Department of Genetics, University Medical Center Groningen, University of Groningen, Groningen, the Netherlands; 4 Department of Preventive Medicine, University of Southern California, Los Angeles, California, USA; 5 Department of Epidemiology of Allergic and Respiratory Diseases, Institut National de la Santé et de la Recherche Médicale (INSERM), Paris, France; 6 ISGlobal, Centre for Research in Environmental Epidemiology (CREAL), Barcelona, Spain; 7 IMIM (Hospital del Mar Medical Research Institute), Barcelona, Spain; 8 Universitat Pompeu Fabra (UPF), Barcelona, Spain; 9 CIBER Epidemiología y Salud Pública (CIBERESP), Barcelona, Spain; 10 European Institute for Systems Biology and Medicine, Université de Lyon, Lyon, France; 11 Centre for Occupational and Environmental Medicine, Stockholm County Council, Stockholm, Sweden; 12 CHU (Centre Hospitalier Universitaire) Montpellier, University of Montpellier, Montpellier, France; 13 Center for Genomic Regulation (CRG), Barcelona Institute of Science and Technology, Barcelona, Spain; 14 Early Origin of the Child’s Health And Development (ORCHAD) team, Centre de Recherche Épidémiologie et Statistique Sorbonne Paris Cité (CRESS-UMR1153) Inserm, Université Paris Descartes, Villejuif, France; 15 The Netherlands Organization for Applied Scientific Research (TNO), Delft, the Netherlands; 16 Generation R Study Group,; 17 Department of Epidemiology, and; 18 Department of Pediatrics, Erasmus MC (Medical Centre), University Medical Center, Rotterdam, the Netherlands; 19 Institute for Risk Assessment Sciences, Utrecht University, Utrecht, the Netherlands; 20 Department of Child and Adolescent Psychiatry/Psychology, Erasmus University Medical Centre–Sophia Children’s Hospital, Rotterdam, the Netherlands; 21 Department of Biosciences and Nutrition, Karolinska Institutet, Stockholm, Sweden; 22 Unit of Chronic Disease Epidemiology, Department of Public Health Epidemiology, Swiss Tropical and Public Health Institute, Basel, Switzerland; 23 University of Basel, Basel, Switzerland; 24 Team of Environmental Epidemiology, Inserm and University Grenoble-Alpes, IAB (U1209), Grenoble, France; 25 Division for Physical and Mental health, Norwegian Institute of Public Health, Oslo, Norway; 26 Department of Women´s and Children´s Health, Karolinska Institutet, Stockholm, Sweden; 27 Division of Intramural Research, National Institute of Environmental Health Sciences, National Institutes of Health, Department of Health and Human Services, Research Triangle Park, North Carolina, USA; 28 Groningen Research Institute for Asthma and COPD (GRIAC), Beatrix Children’s Hospital, Department of Pediatric Pulmonology and Pediatric Allergology, University Medical Center Groningen, University of Groningen, Groningen, the Netherlands; 29 Sachs Children’s Hospital, Stockholm, Sweden

## Abstract

**Background:**

Prenatal exposure to air pollution is considered to be associated with adverse effects on child health. This may partly be mediated by mechanisms related to DNA methylation.

**Objectives:**

We investigated associations between exposure to air pollution, using nitrogen dioxide (NO2) as marker, and epigenome-wide cord blood DNA methylation.

**Methods:**

We meta-analyzed the associations between NO2 exposure at residential addresses during pregnancy and cord blood DNA methylation (Illumina 450K) in four European and North American studies (n = 1,508) with subsequent look-up analyses in children ages 4 (n = 733) and 8 (n = 786) years. Additionally, we applied a literature-based candidate approach for antioxidant and anti-inflammatory genes. To assess influence of exposure at the transcriptomics level, we related mRNA expression in blood cells to NO2 exposure in 4- (n = 111) and 16-year-olds (n = 239).

**Results:**

We found epigenome-wide significant associations [false discovery rate (FDR) p < 0.05] between maternal NO2 exposure during pregnancy and DNA methylation in newborns for 3 CpG sites in mitochondria-related genes: cg12283362 (LONP1), cg24172570 (3.8 kbp upstream of HIBADH), and cg08973675 (SLC25A28). The associations with cg08973675 methylation were also significant in the older children. Further analysis of antioxidant and anti-inflammatory genes revealed differentially methylated CpGs in CAT and TPO in newborns (FDR p < 0.05). NO2 exposure at the time of biosampling in childhood had a significant impact on CAT and TPO expression.

**Conclusions:**

NO2 exposure during pregnancy was associated with differential offspring DNA methylation in mitochondria-related genes. Exposure to NO2 was also linked to differential methylation as well as expression of genes involved in antioxidant defense pathways.

**Citation:**

Gruzieva O, Xu CJ, Breton CV, Annesi-Maesano I, Antó JM, Auffray C, Ballereau S, Bellander T, Bousquet J, Bustamante M, Charles MA, de Kluizenaar Y, den Dekker HT, Duijts L, Felix JF, Gehring U, Guxens M, Jaddoe VV, Jankipersadsing SA, Merid SK, Kere J, Kumar A, Lemonnier N, Lepeule J, Nystad W, Page CM, Panasevich S, Postma D, Slama R, Sunyer J, Söderhäll C, Yao J, London SJ, Pershagen G, Koppelman GH, Melén E. 2017. Epigenome-wide meta-analysis of methylation in children related to prenatal NO2 air pollution exposure. Environ Health Perspect 125:104–110; http://dx.doi.org/10.1289/EHP36

## Introduction

Air pollution exposure has been associated with different types of health effects, such as adverse pregnancy outcomes (Pedersen et al. 2013), childhood airway disease (Minelli et al. 2011), and neurodevelopmental disorders (Calderón-Garcidueñas et al. 2014). Oxidative stress and inflammatory responses have been suggested to be among key pathophysiological mechanisms linking air pollution exposure to the health end points. Even though the molecular processes are not fully understood, there is evidence that air pollution may act partly through epigenetic mechanisms (Gruzieva et al. 2014). Some studies show that DNA methylation, one of the key epigenetic mechanisms, is altered in children exposed to air pollution (Perera et al. 2009; Rossnerova et al. 2013; Tang et al. 2012). A few candidate gene studies have reported differential methylation in genes involved in oxidative stress and chronic inflammation in relation to prenatal (Perera et al. 2009; Tang et al. 2012) and postnatal (Hew et al. 2015; Nadeau et al. 2010; Salam et al. 2012) air pollution exposure. These findings were further supported by animal studies showing that methylation changes within inflammatory genes after exposure to diesel exhaust particles (Liu et al. 2008). Some of these epigenetic modifications were also linked to differential protein expression (Hew et al. 2015). However, genome-wide methylation analyses allowing a hypothesis-free assessment of epigenetic modifications in relation to air pollution exposure are sparse (Jiang et al. 2014; Rossnerova et al. 2013).

Both animal and human studies suggest that exposures affecting epigenetic markers may have a substantial impact if occurring *in utero* (de Planell-Saguer et al. 2014), particularly in light of extensive epigenetic reprogramming during embryogenesis (Cortessis et al. 2012; Wright and Brunst 2013). This has been demonstrated in epigenome-wide studies of methylation in offspring related to maternal smoking during pregnancy (Joubert et al. 2016; Richmond et al. 2015). To our knowledge, no study has evaluated the role of prenatal air pollution exposure on methylation levels across the genome in newborns.

For the present study, we used a large collection of genome-wide DNA methylation data to investigate associations between prenatal exposure to nitrogen dioxide (NO_2_), as an indicator of traffic-related air pollution, and cord blood DNA methylation. In addition, we applied a literature-based candidate approach to evaluate the importance of prenatal NO_2_ exposure for DNA methylation within a set of antioxidant and anti-inflammatory genes. Furthermore, the continuance of associations between maternal exposure to NO_2_ and cord blood DNA methylation changes at key cytosine-guanine dinucleotide sites (CpGs) was examined in a sample of 4- and 8-year-old children, as well as differences in gene expression of selected genes in relation to air pollution exposure.

## Methods

### Study Population

Four studies participating in the Pregnancy and Childhood Epigenetics consortium (PACE) were included in the meta-analysis of NO_2_ exposure during pregnancy and cord blood DNA methylation. These are Mechanisms of the Development of ALLergy (MeDALL), the Generation R Study (the Netherlands), the Children’s Health Study (CHS; USA), and the Mother and Child Cohort Study (MoBa; Norway). MeDALL represents a pooled sample of four cohorts with uniform methylation measurements in paired samples either in cord blood and 4–5 years: Infancia y Medio Ambiente (INMA; Spain) and Etudes des Déterminants pré et postnatals précoces du développement et de la santé de l’ENfant (EDEN; France); or at 4 and 8 years: Children’s Allergy Environment Stockholm Epidemiology study (BAMSE; Sweden) and Prevention and Incidence of Asthma and Mite Allergy (PIAMA; the Netherlands). Two of the MEDALL cohorts with cord blood methylation data (INMA and EDEN) contributed to the meta-analysis on newborns. Methylation data in older children in MeDALL (age 4–5 years for INMA, EDEN, BAMSE, and PIAMA, and age 8 years for BAMSE and PIAMA), as well as an independent methylation data set from the BAMSE cohort at age 8 years (BAMSE EpiGene), the latter consisting of asthma cases and healthy controls (Melén et al. 2013), were used for the subsequent look-up of the findings in cord blood meta-analysis. Information about study design, recruitment, and procedures for data collection in each cohort are provided in the “Materials and Methods” section in the Supplemental Material. Consent for blood sampling was obtained from all parents. Ethics approval for each study was obtained from local authorized review boards.

### Air Pollution Exposure Assessment

In the MeDALL cohorts, the Generation R Study, and BAMSE EpiGene mean concentrations of NO_2_ during pregnancy were estimated at maternal home addresses through land-use regression (LUR) models developed for each study area within the ESCAPE (European Study of Cohorts for Air Pollution Effects) project (Pedersen et al. 2013). LUR models for MoBa were developed following the ESCAPE methodology. In the CHS, air quality monitoring data (Peters et al. 1999) and the U.S. Environmental Protection Agency (EPA) Air Quality System (https://www.epa.gov/aqs) were used to assign estimates of prenatal exposure for NO_2_. Detailed descriptions of exposure assessment are provided in the “Materials and Methods” section in the Supplemental Material.

### Profiling of DNA Methylation

Each cohort independently conducted laboratory measurements and quality control (QC) as described in the “Materials and Methods” section in the Supplemental Material. The samples for each cohort underwent bisulfite treatment using the EZ-96 DNA Methylation kit (Zymo Research Corporation, Irvine, CA, USA), and were subsequently processed with the Illumina Infinium HumanMethylation450 BeadChip (Illumina Inc., San Diego, CA, USA).

Details on QC of samples are provided in the “Materials and Methods” section in the Supplemental Material. Cohorts used validated, published statistical methods for normalizing their methylation data on the untransformed methylation beta values (ranging from 0 to 1), such as “DASEN” (Pidsley et al. 2013), “DASES” (Touleimat and Tost 2012), and BMIQ (Teschendorff et al. 2013). Furthermore, we excluded from the meta-analysis probes that mapped to the X (*n* = 11,232) or Y (*n* = 416) chromosomes, leaving a total of 472,299 CpGs included in the meta-analysis.

Data on mRNA gene expression were available in the BAMSE (239 children 16 years of age) and the INMA (111 children 4 years of age) cohorts through the MeDALL project (Bousquet et al. 2011). Whole blood was collected in PAXGene tubes, and RNA was extracted using PAXgene Blood RNA kit (QIAGEN, Courtaboeuf, France) and assessed for quality. Gene expression data were obtained using Affymetrix HTA 2.0 Genechips (Affymetrix, Inc., Santa Clara, CA, USA). Additional information is provided in the “Materials and Methods” section in the Supplemental Material.

### Statistical Analyses

First, we examined the association between exposure to NO_2_ and methylation levels across the genome using robust linear regression to account for any potential outliers and heteroskedasticity in the data (Fox and Weisberg 2011). Untransformed normalized methylation β-values were used. All included samples were analyzed on a cohort level, except for the pooled MeDALL study with coordinated methylation measurements as well as air pollution exposure assessment according to a harmonized protocol. All analyses were adjusted for an *a priori* selected panel of covariates: sex, maternal smoking during pregnancy, municipality at birth (in BAMSE), cohort-specific batch indicator(s), cohort indicator (in the pooled MeDALL sample set), and ancestry (in CHS). In addition, age at biosampling was included in the analyses of the older children. As a sensitivity analysis we also adjusted for asthma status in the older children analyses. Cohort-specific results of the cord blood EWAS (epigenome-wide association studies) were subsequently included in a fixed-effects meta-analysis (*I* ^2^ random-effects tests for heterogeneity did not display heterogeneity across cohorts) by combining *p*-values across studies, taking into account study-specific weights based on the inverse of the corresponding standard errors (Willer et al. 2010).

DNA methylation sites were annotated based on data provided by Illumina (Bibikova et al. 2011). Because DNA methylation patterns within genetic regions are correlated, we used the false discovery rate (FDR) procedure to account for multiple testing (Strimmer 2008), rather than the more stringent Bonferroni adjustment that assumes independent effects of all CpG sites. CpG sites with FDR < 0.05 threshold were labeled significant.

It has been demonstrated that differences in DNA methylation can arise from variability of cell composition in whole blood (Reinius et al. 2012). To adjust for this, we estimated the fraction of CD8T, CD4T, NK cells, B cells, monocytes, and granulocytes in each sample through the reference-based Houseman method (Houseman et al. 2012) using the estimateCellCounts function in the minfi Bioconductor package in R (Jaffe and Irizarry 2014). We adjusted for cell composition by including the six estimated cell type fractions as covariates in the multivariate linear regression. Additionally, as a sensitivity analysis we applied a new method of cell proportion estimation for cord blood samples in the MeDALL study (Bakulski et al. 2016).

Second, we investigated whether associations between NO_2_ exposure and methylation levels in the top 25 CpGs (corresponding to *p* < 2.59 × 10^–5^) in the cord blood analyses persisted in older children, employing a single CpG look-up approach in available samples of 4-year-olds (pooled MeDALL sample), as well as in 8-year-olds (meta-analyzed pooled MeDALL sample and the BAMSE EpiGene). For these look-up analyses, a CpG with a nominal *p*-value < 0.05 was considered to be statistically significant.

Third, using a candidate-gene approach based on a literature search for air pollution associated genes we investigated separately a set of 739 CpGs in 38 antioxidant and inflammatory genes (*TGFB1, ARG1, ARG2, GSTM1, GSTP1, NQO1, SOD2, GPX1, HMOX-1, CAT, GSTT1, EPHX1, NOS2, TNF, NFE2L2, GSS, GPX7, GPX2, GSTZ1, ALB, SRXN1, NOX5, ALOX12, NCF2, AOX1, MPV17, SIRT2, MBL2, OXSR1, OXR1, NUDT1, DUOX2, EPX, PXDNL, PXDN, MPO, LPO, TPO*) (Carlsten and Melén 2012; Chen et al. 2015; Minelli et al. 2011; Nagiah et al. 2015), by extracting the results from meta-analysis (excluding cg01957222, available only in one cohort). Fourth, to assess functional effects related to methylation profiles, we investigated whether genes annotated to the identified CpGs were differentially expressed in relation to air pollution exposure during pregnancy and at the time of biosampling by means of linear regression analysis. Finally, pathways associated with differentially methylated sites (*p* < 0.0001) were interrogated using ConsensusPathDB database (http://cpdb.molgen.mpg.de) (Kamburov et al. 2013).

Air pollution concentrations were entered as continuous variables without transformation. The results are presented as change in methylation β-value per 10 μg/m^3^ of increase in NO_2_. All study-specific statistical analyses were performed using R (version 3.0.1; R Project for Statistical Computing) and Bioconductor packages (Gentleman et al. 2004), and the meta-analysis was performed using METAL software (Willer et al. 2010). For the most significant results we used the web-based plotting tool CoMet to graphically display additional information about all available CpGs within the same gene including physical location, correlation, and statistical significance (Martin et al. 2015). Cord blood methylation data from the MeDALL samples (*n* = 280) were used to compute the correlations between the CpG sites within selected genes.

## Results

**Figure 1:**
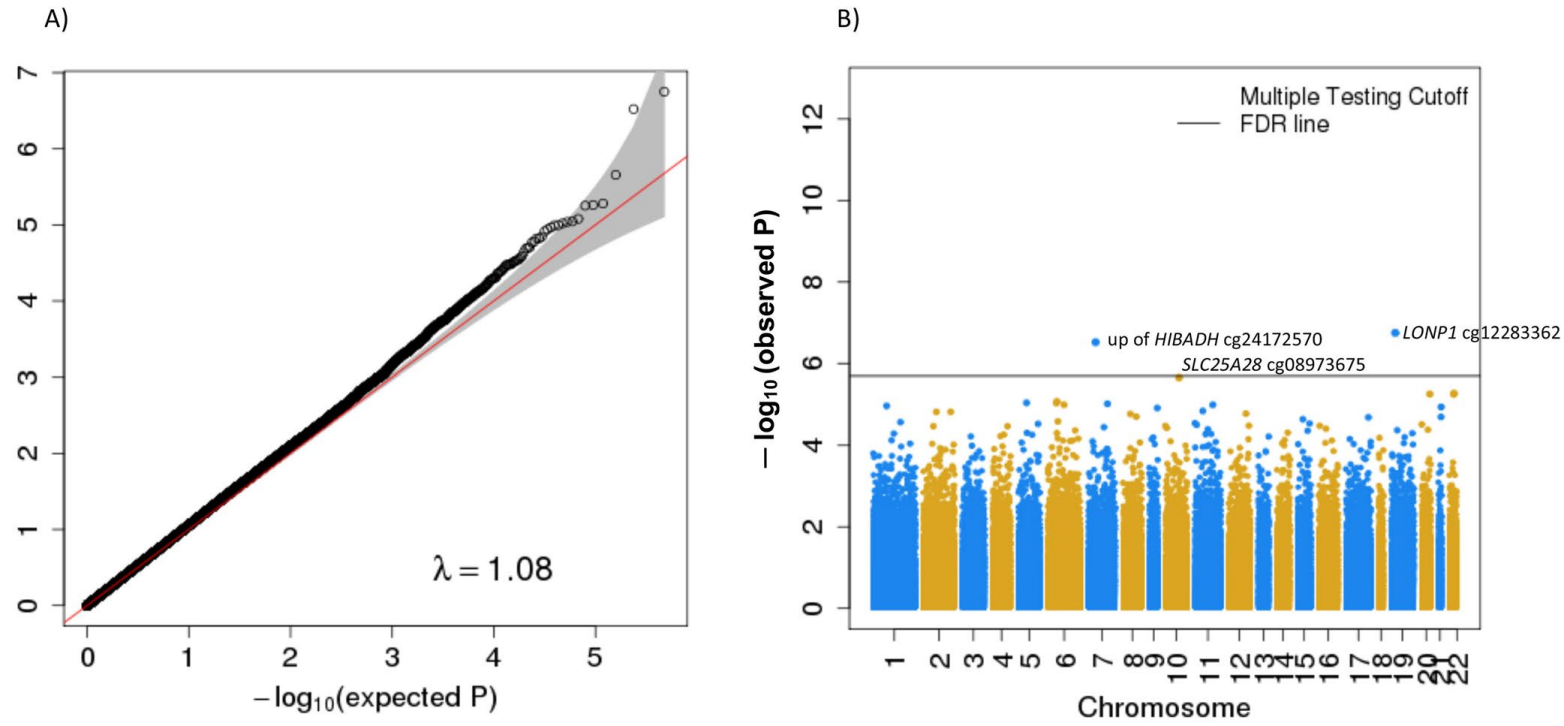
Quantile–quantile plot (*A*) and Manhattan plot (*B*) for epigenome-wide meta-analysis of the association between prenatal NO_2_ exposure and cord blood DNA methylation (*n* = 1,508). (*B*) Three CpGs were considered statistically significant using FDR correction (solid horizontal line): cg12283362 in *LONP1,* cg24172570 3.8 kbp upstream of *HIBADH*, and cg08973675 in *SLC25A28. *

The baseline characteristics of the study population of the original cohorts, and of subjects included in the present analyses are presented in Table S1 and Table 1, respectively. Exposure contrasts, indicated by the interquartile ranges were smallest for the MoBa (5.4 μg/m^3^) and Generation R (5.8 μg/m^3^) cohorts, and highest for the pooled MeDALL sample (28.1 μg/m^3^). In total, 1,508 children were included in the discovery meta-analysis of prenatal NO_2_ exposure and cord blood methylation. Plotted –log10 (*p*-values) from the combined analysis of 472,299 CpGs across the genome in cord blood samples of participants of the MeDALL, Generation R, CHS, and MoBa studies are presented in Figure 1. The quantile–quantile plot did not reveal any significant inflation in the distribution of observed *p*-values (lambda = 1.08). We found epigenome-wide significant associations (FDR *p*-value < 0.05) between NO_2_ exposure and DNA methylation for 3 CpGs, one mapped to *lon peptidase 1* (*LONP1,* cg12283362, chromosome 19), one located 3.8 kbp upstream of the *3-hydroxyisobutyrate dehydrogenase* (*HIBADH,* cg24172570, chromosome 7), and a third mapped to *solute carrier family 25* (*SLC25A28,* cg08973675, chromosome 10) (Table 2). We also observed that methylation levels of these top 3 CpGs significantly changed with NO_2_ exposure levels in a dose-dependent manner with negative trend for cg24172570 (3.8 kbp upstream of *HIBADH*) and cg12283362 (*LONP1*), and positive for cg08973675 (*SLC25A28*) as indicated by the trend test (see Figure S1), although threshold effects or nonlinear associations cannot be completely ruled out. The top hits were largely unaltered by adjustment for predicted cell type components, although cg08973675 (*SLC25A28* chromosome 10) was no longer significant at the FDR significance level (see Figure S2 and Table S2). Interestingly, cg01610636 in *PLVAP* (chromosome 19) encoding plasmalemma vesicle associated protein, as well as cg21022949 located 19.7 kbp downstream of *G-protein-coupled receptor 55* (*GPR55,* chromosome 2) appeared to be FDR-significant after cell-type correction (*p* = 7.0 × 10^–7^ and *p* = 8.9 × 10^–7^ corrected and *p* = 0.002 and 1.5 × 10^–5^ uncorrected, respectively). A sensitivity analysis based on the MeDALL sample set applying a novel adjustment approach for cord blood cells according to Bakulski et al. (2016) showed very good agreement between the results of analyses with and without cell-count adjustment (epigenome-wide correlation of β-coefficients = 0.95, and *p*-values = 0.82; see Table S3). In addition, we checked the potential influence of outliers on our top hits results in the MeDALL sample set by trimming outliers defined by > 3 interquartile ranges below the first quartile or above the fourth quartile (NIST 2013). After outlying CpGs have been excluded by the trim, we re-ran the analyses and got essentially unchanged results (data not shown).

**Table 1:** Characteristics for individuals of the included cohorts.

Characteristic	Birth				4–5 years	8 years	
	MeDALL pooled^*a*^ EDEN (France), INMA (Spain) (*n* = 280)	Generation R (the Netherlands) (*n* = 809)	CHS (USA) (*n* = 226)	MoBa (Norway) (*n* = 193)	MeDALL pooled^*a*^ BAMSE (Sweden), EDEN (France), INMA (Spain), PIAMA (the Netherlands) (*n* = 733)	MeDALL pooled^*a*^ BAMSE (Sweden), PIAMA (the Netherlands) (*n* = 444)	BAMSE EpiGene (Sweden) (*n* = 342)
NO_2_ during pregnancy (μg/m^3^): percentiles 25th, 50th, 75th (minimum–maximum)	19.0, 37.5, 47.1 (9.0–89.9)	36.0, 38.7, 41.8 (28.6–55.9)	23.0, 32.1, 36.9 (7.5–51.0)	7.5, 10.3, 12.9 (0.01–27.6)	20.2, 31.2, 40.4 (9.0–89.9)	19.7, 27.3, 35.4 (9.9–59.8)	17.9, 23.3, 33.3 (9.3–58.7)
Annual NO_2_ at the current address at the time of biosampling (μg/m^3^): percentiles 25th, 50th, 75th (minimum–maximum)	—	—	—	—	11.4, 21.1, 23.7 (2.6–95.6)	9.1, 14.1, 22.5 (6.1–39.7)	8.1, 9.4, 13.1 (6.0–29.1)
Male sex [*n* (%)]	155 (55.4)	427 (52.8)	93 (41.2)	101 (52.3)	398 (54.3)	230 (51.8)	181 (52.9)
Age at biosampling (years): mean ± SD (minimum–maximum)	—	—	—	—	4.4 ± 0.5 (3.3–6.0)	8.2 ± 0.4 (7.3–9.7)	8.3 ± 0.5 (7.4–10.5)
Maternal smoking during pregnancy [*n* (%)]	48 (17.1)	200 (24.7)	14 (6.2)	15 (7.8)	101 (13.8)	52 (11.7)	41 (12.0)
^***a***^In the MeDALL sample, methylation data measured in cord blood are available in EDEN (*n *= 93) and INMA (*n *= 187); at 4–5 years available in EDEN (*n *= 82), INMA (*n *= 195), BAMSE (*n *= 232), and PIAMA (*n *= 224); at 8 years available in BAMSE (*n *= 243) and PIAMA (*n *= 201).							

**Table 2:** Top 25 CpGs from the epigenome-wide meta-analysis of the association between prenatal NO_2_ exposure and newborn cord blood DNA methylation (*n* = 1,508 newborns from MeDALL, Generation R, CHS and MoBa cohorts).

Chr	Position (build 37)	CpG	Mapped gene	Gene group	Coef	SE	*p*-Value	Direction
19	5709149	cg12283362	*LONP1*^*FDR,a*^	Body	–0.007	0.0014	1.78 × 10^–7^	–??–^*b*^
7	27561178	cg24172570	*HIBADH*^*FDR,a,c*^		–0.004	0.0008	3.01 × 10^–7^	––?–^*b*^
10	101380289	cg08973675	*SLC25A28*^*FDR,a*^	TSS200	0.005	0.0011	2.20 × 10^–6^	++++
22	40355732	cg17988310	*GRAP2*	Body	0.004	0.0009	5.25 × 10^–6^	++++
20	61427684	cg14582546	*C20orf20*	TSS200	0.005	0.0011	5.50 × 10^–6^	++++
22	39323510	cg12276768	*APOBEC3A*^*c*^		0.003	0.0006	5.60 × 10^–6^	++++
6	30688588	cg21660604	*TUBB*	Body	0.002	0.0003	8.36 × 10^–6^	++++
5	77284206	cg26815688	*AP3B1*^*c*^		–0.002	0.0005	9.03 × 10^–6^	––––
6	30524763	cg03860665	*PRR3;GNL1*	5’UTR; 1stExon	0.002	0.0005	9.17 × 10^–6^	++++
7	117824040	cg08301459	*NAA38*	TSS200	0.002	0.0003	9.58 × 10^–6^	++?+
6	33359817	cg04757012	*KIFC1*	Body	0.001	0.0003	1.01 × 10^–5^	++++
11	74871202	cg12537437	*SLCO2B1*	Body; 5’UTR	–0.004	0.0008	1.02 × 10^–5^	–––+
1	35226135	cg01828548	*GJB4*	5’UTR	–0.005	0.0011	1.08 × 10^–5^	–––+
21	46032086	cg26386968	*C21orf29;KRTAP10-8*	Body; 1stExon	–0.007	0.0015	1.15 × 10^–5^	––?–
9	139607421	cg12657416	*FAM69B*	Body	0.103	0.0236	1.22 × 10^–5^	?+?+
11	34460856	cg03728580	*CAT*	Body	0.003	0.0007	1.43 × 10^–5^	++++
2	231809697	cg21022949	*GPR55*^*c*^		0.001	0.0002	1.51 × 10^–5^	++++
2	98409069	cg06840305	*TMEM131*	Body	–0.002	0.0004	1.51 × 10^–5^	––––
12	120967065	cg11075121	*COQ5*	TSS200	0.002	0.0004	1.66 × 10^–5^	++++
8	48099615	cg03271173	*IGLV8OR8-1*^*c*^		–0.003	0.0006	1.70 × 10^–5^	––+–
8	110346503	cg25407888	*ENY2;NUDCD1*	TSS200	0.003	0.0006	1.98 × 10^–5^	++++
21	45753677	cg24316255	*C21orf2*	Body	–0.003	0.0006	2.00 × 10^–5^	–––+
17	78851213	cg08314949	*RPTOR*	Body; Body	0.013	0.0031	2.06 × 10^–5^	+–?+
15	59063272	cg01889112	*FAM63B*	TSS200; TSS200	0.002	0.0004	2.29 × 10^–5^	++++
6	31382102	cg26504614	*MICA*	Body	–0.005	0.0011	2.59 × 10^–5^	–?––
Shown are top 25 CpGs ordered by *p*-value. Results presented per 10 μg/m^3^ increase in prenatal NO_2_ exposure. Column heads: Chr: chromosome; Position: chromosomal position based on NCBI human reference genome assembly build 37; Mapped Gene: UCSC annotated gene; Gene group: UCSC gene region feature category; Coef: regression coefficient; SE: standard error for regression coefficient; Direction: direction of effect across cohorts included in the statistical model (MeDALL, Generation R, CHS, and MoBa): NO_2_ exposure during pregnancy was associated with increased (+) or decreased (–) methylation, or missing (?) result. ^***a***^Genome-wide significance threshold (FDR *p* < 0.05). ^***b***^Data on methylation for cg12283362 were available in 473 individuals, and for cg24172570 in 1,282 individuals. ^***c***^cg24172570 is located 3.8 kbp upstream of *HIBADH; cg12276768 *is located* 25.2* kbp upstream of *APOBEC3A;* cg26815688 is located 21.1 kbp upstream of *AP3B1;* cg21022949 is located 19.7 kbp downstream of *GPR55*; cg03271173 is located *14.5* kbp upstream of *IGLV8OR8-1.*								

We further investigated whether these three associations (cg24172570 3.8 kbp upstream of *HIBADH,* cg12283362 *LONP1* and cg08973675 *SLC25A28*) between air pollution exposure and methylation at birth persisted later in childhood. We observed similar significant change in methylation level of cg08973675 (*SLC25A28*) in all available samples of the 4-year-old children of the MeDALL study (*p* = 0.03), as well as of the 8-year-olds of the meta-analyzed MeDALL and BAMSE EpiGene samples (*p* = 0.04), in relation to prenatal NO_2_ exposure (see Table S4). None of the other two top hits could be replicated in the older children. In addition, because the MeDALL sample set of 4-year-olds included two cohorts with paired samples (cord blood and 4 years), EDEN and INMA, we re-ran the look-up analysis in 4-year-olds separately in these two cohorts. A significant change in methylation of cg08973675 (*SLC25A28*) associated with NO_2_ exposure during pregnancy was seen in the combined EDEN and INMA samples (*p* = 0.005), providing further evidence of the persistence of the association between air pollution exposure and methylation at birth into older age (see Table S5). The results remained unchanged after additional adjustment for asthma status (data not shown).

Among CpGs of selected antioxidant defense genes previously linked to air pollution exposure, 2 CpGs in *catalase* gene (*CAT* cg03728580 and cg17034036, chromosome 11), as well as 1 in *thyroid peroxidase* gene (*TPO* cg01385533, chromosome 2) were differentially methylated (FDR *p* < 0.05) (see 10 top significant CpGs in Table 3 and all nominally significant CpGs in Table S6). In addition, 4 of 15 available CpGs in the *CAT*, as well as 9 of 87 CpGs in the *TPO* were differentially methylated at the nominal significance level (*p* < 0.05) (see Tables S7 and S8). In the analyses in older children, methylation changes in cg01385533 (*TPO*) in 4-year-olds were found to be of similar direction as in the newborns in relation to annual NO_2_ exposure at the time of biosampling (*p* = 0.04), as well as in 8-year-olds in relation to prenatal exposure (*p* = 0.04) (see Table S9). Associations did not persist to older ages for the *CAT* probes. We found some evidence for localized clustering around the top FDR-significant CpGs in *CAT* and *TPO*, with moderate co-methylation within the *CAT* region but weak in the *TPO* (see Figure S3).

**Table 3:** Top 10 significant CpGs within oxidative stress genes extracted from the epigenome-wide meta-analysis of the association between prenatal NO_2_ exposure and newborn cord blood DNA methylation (*n* = 1,508 newborns from MeDALL, Generation R, CHS, and MoBa cohorts).

Chr	Position (build 37)	CpG	Mapped gene	Gene group	Coef	SE	*p*-Value	Direction
11	34460856	cg03728580	*CAT*^*FDR*^	Body	0.003	0.001	0.00001	++++
11	34461028	cg17034036	*CAT*^*FDR*^	Body	0.002	0.001	0.0001	++++
2	1482597	cg01385533	*TPO*^*FDR*^	Body	–0.003	0.001	0.0004	–?––
1	226023590	cg05935800	*EPHX1*	Body	–0.002	0.001	0.002	––––
20	33539306	cg13607138	*GSS*	Body	–0.003	0.001	0.003	––?–
8	107642385	cg17526936	*OXR1*	Body	–0.002	0.001	0.004	––?–
2	1544120	cg19407717	*TPO*	Body	–0.002	0.001	0.004	––––
2	1479523	cg13703866	*TPO*	Body	–0.001	0.000	0.005	––––
11	34460336	cg07768201	*CAT*	TSS200	0.003	0.001	0.006	++++
1	226012507	cg03337430	*EPHX1*	TSS1500;5’UTR	0.001	0.000	0.006	+–++
Shown are the top 10 CpGs ordered by *p*-value. Three CpGs were statistically significant using genome-wide significance threshold (FDR *p* < 0.05). Results presented per 10 μg/m^3^ increase in prenatal NO_2_ exposure. Column heads: Chr: chromosome; Position: chromosomal position based on NCBI human reference genome assembly Build 37. Mapped Gene: UCSC annotated gene; Gene group: UCSC gene region feature category; Coef: regression coefficient; SE: standard error for regression coefficient; Direction: direction of effect across cohorts included in the statistical model (MeDALL, Generation R, CHS, and MoBa): NO_2_ exposure during pregnancy associated with increased (+) or decreased (–) methylation, or missing (?) result.								

In functional analysis of available expression data from the 16-year-olds in the BAMSE cohort and the 4-year-olds in the INMA cohort, no significant association of *in utero* NO_2_ exposure with gene expression was detected for any of the studied probes (data not shown). However, current NO_2_ exposure at 16 years was significantly associated with *LONP1*, *CAT*, and *TPO* expression levels in peripheral blood cells of the BAMSE children (Table 4). The results were robust to additional adjustment for measured cell counts. In the INMA cohort, *TPO* and *GPR55* were also significantly differentially expressed in relation to current NO_2_ exposure at 4 years after adjustment for cell counts (*p* < 0.05), although the direction of change differed compared to that in BAMSE.

**Table 4:** Associations between current NO_2_ exposure and gene expression levels in the children of the BAMSE (*n* = 239) and INMA (*n* = 111) cohorts.

Gene	Cohort	LogFC^*a*^	*p*-Value
*TPO*	BAMSE 16 years	0.038	0.032
	INMA 4 years	–0.028	0.004
*CAT*	BAMSE 16 years	–0.098	0.042
	INMA 4 years	0.014	0.660
*LONP1*	BAMSE 16 years	0.034	0.008
	INMA 4 years	–0.007	0.372
*SLC25A28*	BAMSE 16 years	0.003	0.829
	INMA 4 years	–0.0003	0.968
*PLVAP*	BAMSE 16 years	–0.071	0.142
	INMA 4 years	0.002	0.954
*GPR55*	BAMSE 16 years	0.027	0.180
	INMA 4 years	–0.033	0.003
Results presented per 10-μg/m^3^ increase in NO_2_ exposure current with biosampling in the BAMSE and INMA cohorts. LogFC, logarithm fold-change (1 unit of the logFCs translates to a 2-fold change in expression). In the INMA cohort, cell count estimation using expression data was performed using R package CellMix and Abbas data set. Actual cell counts in BAMSE were used. ^***a***^Adjusted for sex, age, municipality at birth (only in BAMSE), maternal smoking during pregnancy, and cell composition.			

Finally, to identify plausible pathways associated with air pollution exposure, we also performed gene set enrichment analysis based on CpGs significantly associated with prenatal NO_2_ in the meta-analysis using an arbitrary cut-off of *p* < 0.0001. A total of 71 unique gene identifiers were entered in the ConsensusPathDB database of which 58 matched. Using FDR *p* < 0.05, a few enriched pathways were identified including “negative regulation of cellular process” (GO term GO:0048523, FDR *p* = 0.04), “negative regulation of biological process” (GO:0048519, FDR *p* = 0.04), and the “integrin-linked kinase signaling” pathway (FDR *p* = 0.02).

## Discussion

This study represents a large-scale epigenome-wide meta-analysis evaluating the association between prenatal air pollution exposure and DNA methylation in newborns. The combined results show suggestive evidence for associations of NO_2_ exposure during pregnancy with methylation differences in several genes, involved in mitochondria function, providing a potential epigenetic biomarker of *in utero* exposure that persisted in early childhood. Using a hypothesis-based approach, we also identified a link between prenatal NO_2_ exposure and methylation of CpG loci in antioxidant enzyme genes, such as *CAT* and *TPO*. Furthermore, we observed differential expression of these two genes in relation to recent exposure to NO_2_.

The three differentially methylated CpG sites—cg12283362 in *LONP1,* cg24172570 3.8 kbp upstream of *HIBADH*, and cg08973675 in *SLC25A28*—represent novel associations in the context of air pollution exposure. The top significant cg12283362 localizes to the gene *LONP1* encoding a protein that belongs to the Lon family of ATP-dependent proteases and mediates the selective degradation of misfolded, unassembled or oxidatively damaged polypeptides in the mitochondrial matrix (Pinti et al. 2015). However, cg12283362 did not pass the QC filter in two of the cohorts with cord blood samples (*n* = 1,035), and the results should therefore be interpreted with caution. The second significant site, cg24172570, was located 3.8 kbp upstream of *HIBADH* that encodes a protein playing a critical role in the catabolism of l-valine. The third one, cg08973675, is annotated to the *SLC25A28* coding for a mitochondrial iron transporter protein that mediates iron uptake. *SLC25A28* was the only top gene with persistent prenatal NO_2_–methylation associations in older children. Interestingly, all three genes are involved in mitochondria function, and mitochondria are known to play an important role in several key pathways of cellular responses to environmental stressors, including response to reactive oxygen species (ROS), nutrient and ATP sensing, and DNA damage response (Shaughnessy et al. 2014).

Recent studies demonstrated that air pollution exposure during pregnancy is associated with changes in global DNA methylation in cord blood cells and placental tissue sampled from the fetal side (Herbstman et al. 2012; Janssen et al. 2013). Global methylation, however, represents the overall methylation state of the genome without indicating which genomic locations are methylated. A study conducted in schoolchildren suggested an impact of air pollution exposure on the DNA methylation patterns in genes related to the immune system, DNA–protein binding, and metabolism of xenobiotics as measured by the Illumina 27K platform (Rossnerova et al. 2013).

We also compared the methylation status at a candidate gene level for genes previously implicated in biologic response to air pollution using a hypothesis-based approach. Oxidative stress and inflammation have been hypothesized as the main mechanisms through which ambient air pollution can affect human health (Esposito et al. 2014). Both experimental and observational studies demonstrate the capacity of NO_2_ along with other air pollutants to activate oxidant pathways through formation of ROS, triggering inflammation and cell death (Lodovici and Bigagli 2011). Studies in human bronchial epithelial cells showed differential expression of genes involved in response to oxidative stress following air pollution exposure (Rossner et al. 2015; Zhou et al. 2015). In our study we observed differential methylation in *CAT* and *TPO*. *CAT* encodes catalase, an antioxidant that catalyzes degradation of hydrogen peroxide and plays a crucial role in protecting cells against ROS. However, long-term exposure to ROS may downregulate *CAT* expression via hypermethylation of a CpG island (Min et al. 2010), which would be in line with our results. Even though DNA methylation and gene expression were measured at different ages, we observed increased methylation in CpGs of the *CAT* gene in newborns together with decreased gene expression in adolescents of the BAMSE study in relation to current NO_2_ exposure at 16 years. This observed pattern of increased methylation and decreased gene expression by NO_2_ exposure is in the expected direction (i.e., the higher the methylation, the lower the gene expression). Furthermore, additional pathway analysis demonstrated that *CAT* was significantly enriched in several gene ontology terms. Thyroid peroxidase, originally described as thyroid specific enzyme, has also been identified in human airway epithelial cells as the only peroxidase differentially expressed in severe asthmatics, thus distinguishing them from healthy controls and milder asthma cases (Voraphani et al. 2014). A recent functional study indicated significantly higher expression of *TPO* in peripheral lymphocytes in pregnant women residing in a highly industrialized area (Nagiah et al. 2015). We also observed decreased methylation in the *TPO* gene in newborns as well as in older children together with differential *TPO* expression in both the BAMSE and the INMA cohorts, although with the opposite direction. Relatively small sample sizes, differences in age, as well as in other exposures might have contributed to the observed difference in the direction of effects; therefore, these results should be interpreted with caution. Furthermore, the present analysis does not involve the possible various isoforms of the genes. Thus, future studies need to assess whether different isoforms are expressed in response to air pollution.

One challenge of genome-wide DNA methylation analyses in blood samples with a mixed cell composition is the difference in methylation patterns between different cell types. In the present analyses we used the reference data for adult peripheral blood to correct for cell type proportions in the cord blood analyses (Reinius et al. 2012). A sensitivity analysis in one of the included studies that applied a new cell type referenced by Bakulski et al. (2016), which takes cord blood cell composition into account, further supported robustness of the results. Although no major differences were detected in the top results with cell-type correction, cg01610636 in *PLVAP* and cg21022949 located 19.7 kbp downstream of *GPR55* appeared to be FDR-significant after cell-type adjustment (according to the Houseman method). Interestingly, *PLVAP* is known to be involved in leukocyte transendothelial cell migration (Keuschnigg et al. 2009). *GPR55* has been implicated as cannabinoid receptor (Ryberg et al. 2007). Further functional analysis did not reveal any difference in expression profiles of *PLVAP* in relation to NO_2_ exposure, but weak associations with *GPR55* expression were observed in the INMA study. Tissue specificity is another potential limitation that may complicate the assessment of epigenetic patterns relevant for air pollution exposure (Bakulski and Fallin 2014). Therefore, using other biological samples, such as airway epithelium or placenta, in future studies may identify important methylation differences in the primary tissues.

The comprehensive evaluation of genome-wide DNA methylation using the Illumina 450K BeadChip together with air pollution exposure information on individual level, as well as availability of samples at multiple ages, are major strengths of this study. All cohort-specific analyses were conducted according to the same analytical protocol. However, the between-cohort differences in statistical methods applied for the quality control, normalization, and adjustment for technical variation may to some extent contribute to diluting of possible associations. A recently published EWAS meta-analysis including the same cohorts reported very robust results in relation to different data processing methods used across the cohorts for normalization and corrections for technical variables such as batch (Joubert et al. 2016). It is also important to note that our analyses were based mainly on Caucasian populations, and it remains to be investigated whether the findings can be extrapolated to other ethnic groups.

We used NO_2_ as a marker of traffic-derived combustion pollutants. Road traffic is considered to be the principal outdoor source of nitrogen dioxide (WHO 2010). Previous measurement studies around roadways have shown that traffic-related pollutants are characterized well by NO_2_, as indicated by high correlations (*r* ~ 0.7–0.96) between measurements of NO_2_ and PM_2.5_ (particulate matter ≤ 2.5 μm), ultrafine particles, and black carbon (Beckerman et al. 2008), including increases in benzene and polycyclic aromatic hydrocarbons (Karner et al. 2010). A potential limitation of the exposure assessment is that the modeled individual concentrations account only for outdoor air pollution at residential addresses and therefore are not equivalent to personal exposure. Indoor exposure and time–activity patterns may introduce some bias, although this will most likely be nondifferential and thus would generally tend to attenuate the associations. Furthermore, several measurement studies conducted in different areas have demonstrated that indoor and outdoor NO_2_ levels are strongly correlated (*R* ^2^ = 0.7–0.9), pointing to indoor NO_2_ concentrations being largely affected by outdoor sources (El-Hougeiri and El Fadel 2004; Verriele et al. 2015; Wichmann et al. 2010).

## Conclusions

Our epigenome-wide meta-analysis provides evidence of cord blood methylation differences in several mitochondria-related genes, in relation to air pollution exposure during pregnancy. Our study also contributes to further understanding of potential underlying mechanisms of the negative health effects of air pollution by highlighting the implications of DNA methylation in several candidate genes involved in antioxidant defense pathways, such as *CAT* and *TPO*.

## Supplemental Material

(1.7 MB) PDF
