# Supplementary material for: Epigenome-Wide Meta-Analysis of Methylation in Children Related to Prenatal NO2 Air Pollution Exposure
Source: Environ Health Perspect. 2016 Jul 22;125(1):104–10. doi: 10.1289/EHP36 (PMC5226705; doi:10.1289/EHP36)
Supplement: (1.7 MB) PDF [file EHP36.s001.acco.pdf]

Environ Health Perspect
DOI: 10.1289/EHP36

Note to readers with disabilities: *EHP* strives to ensure that all journal content is accessible to all readers. However, some figures and Supplemental Material published in *EHP* articles may not conform to 508 standards due to the complexity of the information being presented. If you need assistance accessing journal content, please contact ehp508@niehs.nih.gov. Our staff will work with you to assess and meet your accessibility needs within 3 working days.

Supplemental Material

Epigenome-Wide Meta-Analysis of Methylation in Children Related to Prenatal NO_2 Air Pollution Exposure

Olena Gruzieva, Cheng-Jian Xu, Carrie V. Breton, Isabella Annesi-Maesano, Josep M. Antó, Charles Auffray, Stéphane Ballereau, Tom Bellander, Jean Bousquet, Mariona Bustamante, Marie-Aline Charles, Yvonne de Kluizenaar, Herman T. den Dekker, Liesbeth Duijts, Janine F. Felix, Ulrike Gehring, Mònica Guxens, Vincent V.W. Jaddoe, Soesma A. Jankipersadsing, Simon Kebede Merid, Juha Kere, Ashish Kumar, Nathanael Lemonnier, Johanna Lepeule, Wenche Nystad, Christian Magnus Page, Sviatlana Panasevich, Dirkje Postma, Rémy Slama, Jordi Sunyer, Cilla Söderhäll, Jin Yao, Stephanie J. London, Göran Pershagen, Gerard H. Koppelman, and Erik Melén

Table of Contents

Materials and Methods
Table S1. Characteristics of the included cohorts with available cord blood samples.
Figure S1. Plot of the cumulative distribution function for methylation intensities for the three top-ranked CpG sites.
Figure S2. Quantile-quantile plot (A) and Manhattan plot (B) for epigenome-wide meta-analysis of the association between NO_2 exposure during pregnancy and cord blood DNA methylation, additionally adjusted for cell type composition.
Table S2. Top 25 CpGs from the epigenome-wide meta-analysis of the association between NO_2 exposure during pregnancy and cord blood DNA methylation additionally adjusted for cell composition (n=1,508 newborns from MeDALL, Generation R, CHS and MoBa cohorts).

Table S3. Top 25 CpGs from the analysis of the association between NO_2 exposure during pregnancy and cord blood DNA methylation without adjustment for cell composition (left-side part) and with adjustment for cell composition according to Bakulski (right-side) (n=280 newborns from the MeDALL study).
Table S4. Look-up in older children for top 25 findings for prenatal NO_2 exposure in relation to methylation in meta-analysis of newborns.
Table S5. Look-up in 4-year-old children with paired cord blood and 4-year-old samples for top 25 findings for prenatal NO_2 exposure in relation to methylation in meta-analysis of newborns.
Table S6. Nominally significant CpGs within oxidative stress genes extracted from the epigenome-wide meta-analysis of the association between prenatal NO_2 exposure and newborn cord blood DNA methylation (n=1,508 newborns from MeDALL, Generation R, CHS and MoBa cohorts).
Table S7. All available CpGs mapped to *CAT* gene from the epigenome-wide meta-analysis of the association between NO_2 exposure during pregnancy and newborn cord blood DNA methylation (N=1,508 newborns from MeDALL, Generation R, CHS and MoBa cohorts).
Table S8. All available CpGs mapped to *TPO* gene from the epigenome-wide meta-analysis of the association between NO_2 exposure during pregnancy and newborn cord blood DNA methylation (N=1,508 newborns from MeDALL, Generation R, CHS and MoBa cohorts).
Table S9. Look-up in older children for top CpGs in the *CAT* and *TPO* genes for prenatal NO_2 exposure in relation to methylation in meta-analysis of newborns (n=1,508).
Figure S3. Regional plots and co-methylation patterns for the genes containing FDR-significant EWAS CpG sites.

Acknowledgments

Funding

Materials and Methods

MeDALL

MeDALL (Mechanisms of the Development of ALLergy) is a collaborative project supported by the European Union under the Health Cooperation Work Programme of the 7th Framework programme (grant agreement number 261357) (Bousquet et al. 2011). MeDALL epigenetics studies include four birth cohorts. These are EDEN, BAMSE, PIAMA and INMA.

MeDALL – EDEN

The EDEN (Etude des Déterminants pré et post natals du développement et de la santé de l'Enfant) study is a prospective Birth Cohort Study (https://eden.vjf.inserm.fr/), which has been described in detail elsewhere (Heude et al. 2015). Pregnant women seen for a prenatal visit at the departments of Obstetrics and Gynecology of the University Hospital of Nancy and Poitiers before their twenty-fourth week of amenorrhea were invited to participate. Enrolment started in February 2003 in Poitiers and September 2003 in Nancy; it lasted 27 months in each centre. Among eligible women, 55% (2002 women) accepted to participate. The study has been approved by the ethical committees « Comité Consultatif pour la Protection des Personnes dans la Recherche Biomédicale », Le Kremlin-Bicêtre University hospital, and « Commission Nationale de l'Informatique et des Libertés ».

MeDALL - INMA

The INMA—INfancia y Medio Ambiente—(Environment and Childhood) Project is a network of birth cohorts in Spain that aim to study the role of environmental pollutants in air, water and diet during pregnancy and early childhood in relation to child growth and development (Guxens et al. 2012). The study has been approved by Ethical Committee of each participating centre and written consent was obtained from participating parents. Data for this study came from INMA Sabadell cohort (children born between 2004 and 2007). A total of 187 mothers with prenatal air pollution information and offspring cord blood DNA methylation were included, as well as 195 samples at age 4 years.

MeDALL - PIAMA

For the PIAMA birth cohort study, pregnant women were recruited in 1996-1997 during their second trimester of pregnancy from a series of communities in the North, West, and Centre of The Netherlands as described elsewhere (Wijga et al. IJE 2014). Non-allergic pregnant women were invited to participate in a "natural history" study arm. Pregnant women identified as allergic through a validated screening questionnaire were primarily allocated to

an intervention arm with a random subset allocated to the natural history arm. The intervention involved the use of mite-impermeable mattress and pillow covers. The study started with 3,963 newborns. Information on the children's health, socio-demographic and lifestyle factors as well as residential characteristics was collected by questionnaire annually until age 8 years and then at ages 11 and 14 years.

MeDALL – BAMSE

BAMSE is a prospective population-based cohort study of children recruited at birth and followed during childhood and adolescence. Details of the study design, inclusion criteria, enrolment and data collection are described elsewhere (Thacher et al. 2016; Wickman et al. 2002). In short, 4,089 children born between 1994 and 1996 in four municipalities of Stockholm County were enrolled. At baseline, when the infant was approximately 2 months of age, parents completed a questionnaire that assessed residential characteristics, as well as socioeconomic and lifestyle factors. When children were 1, 2, 4, 8, 12 and 16 years, the parents completed questionnaires focusing on children's symptoms related to wheezing and allergic diseases, as well as various exposures. The survey response rates were 96%, 94%, 91%, 84%, 82% and 78%, respectively. Furthermore, blood was obtained at ages 4, 8 and 16 years from 2,605 (63.7%), 2,470 (60.4%) and 2,547 (62.2%) children, respectively. The baseline and follow-up studies were approved by the Regional Ethical Review Board, Karolinska Institutet, Stockholm, Sweden, and the parents of all participating children provided informed consent.

MeDALL DNA methylation measurements

In the MeDALL study, peripheral blood samples were collected from all consenting cohort participants, and DNA from peripheral and cord blood samples was extracted using the QIAamp blood kit (Qiagen, Inc, Valencia, CA) or equivalent protocols, followed by a precipitation-based concentration using GlycoBlue (Ambion, Austin, Tex). DNA concentration was determined by Nanodrop measurement and picogreen quantification. 500 ng of DNA were bisulfite-converted using the EZ 96-DNA methylation kit following the manufacturer's standard protocol, and DNA methylation measured using the Illumina Infinium HumanMethylation450 beadchip (Illumina, Inc., San Diego, USA). DNA methylation data were preprocessed using the Minfi package (Aryee et al. 2014).

In quality control, samples that did not provide significant methylation signals in more than 10% of probes (detection p-value=0.01) were regarded as bad quality samples and were directly removed. In addition, samples were excluded in case of low staining efficiency, low single base extension efficiency, low stripping efficiency of DNA from probes after single base extension, poor hybridization performance, poor bisulfite conversion and high negative control probe staining. Moreover, we used 65 SNP probes to check for concordances between paired DNA samples from the sample individual and assessed the methylation distribution of X-chromosome to verify gender. Paired samples which show Pearson correlation coefficient <0.9 were regarded as sample mixed ups and were excluded from the study. Furthermore, we excluded probes on sex chromosomes, probes that mapped on multi-loci, the 65 random SNPs assay, and probes that contained SNP(s) at the target CpG sites with a minor allele frequency >10% . A series of steps were completed for quality control and data analysis. First, we implemented sample filtering to remove bad quality and mixed up samples. Second, we filtered out the probes to remove the CpG sites which are not mapped to unique location on the genome and CpGs containing single nucleotide polymorphisms (SNPs) at the target site. Third, we implemented "DASEN" (Pidsley et al. 2013) to perform signal correction and normalization.

To remove bias in methylation profiles unrelated to underlying biological processes, we implemented a correction procedures based on 613 negative control probes presented in 450K arrays since these negative control probes are supposed to not relate to biological variation. Finally, we implemented principal component analysis (PCA) on control probes data, then, we performed 10000 permutation for controls probes data and selected principal components with p-value defined as to get the p-value of (number of var(random pc) >var(pc)) / (number of permutations) $<10^{-4}$. The methylation data for each CpG are thus the residuals from a linear model fitting incorporating the significant 5 PCs.

MeDALL gene expression measurements

Data on mRNA gene expression were available in the BAMSE (239 children aged 16 years) and the INMA (111 children aged 4 years) cohorts. Whole blood was collected in PAXGene tubes and RNA was extracted using PAXgene Blood RNA kit (QIAGEN, Courtaboeuf, France). Quantity of extracted RNA and quality assessment were performed with Dropsense96 (Trinean, Gentbrugge, Belgium) and Tapestation (Agilent, Les Ulis, France) instruments, respectively, discarding 3 samples (BAMSE n=2, INMA n=1). RNA of

highest quality was selected for amplification, labeling and hybridization on Affymetrix HTA 2.0 Genechips using Affymetrix IVT kit (Affymetrix, Inc. USA) at the European Institute for Systems Biology and Medicine in Lyon. Data were then processed at the probesets level for RMA normalization using Expression Console Software from Affymetrix v1.4. Expression transcripts were annotated using version 35 of Affymetrix annotation. In the INMA cohort, 12 samples were excluded because they were outliers defined as more than 3SD from the mean for PC1 or PC2 (n=8) or there were sex discrepancies (n=8). The Affy HTA 2.0 technology we used is composed of 428, 250, 328, 242, 80 and 126 probes for the *TPO, CAT, LONP1, SLC25A28, PLVAP* and *GPR55,* respectively. Each of these sets contains probes that are scattered at various localization along the transcripts, including exons and junctions, averaged into one Transcript Cluster (per gene) summarizing the specific gene expression level. For the BAMSE samples, automated cell count was obtained by flow cytometry performed at the Karolinska University Laboratory in Stockholm, Sweden.

BAMSE EpiGene

In the BAMSE cohort we also had an independent methylation dataset. Epigenome-wide DNA methylation was measured in 472 Caucasian children, using DNA extracted from blood samples collected at the age of 8 years. An aliquot (500 ng) of DNA per sample underwent bisulfite conversion using the EZ-96 DNA Methylation kit (Zymo Research Corporation, Irvine, USA). Samples were plated onto 96-well plates in randomized order. Samples were processed with the Illumina Infinium HumanMethylation450 BeadChip (Illumina Inc., San Diego, USA).

Quality control of analysed samples was performed using standardized criteria. Samples were excluded in case of sample call rate <99%, colour balance >3, low staining efficiency, poor extension efficiency, poor hybridization performance, low stripping efficiency after extension and poor bisulfite conversion. We also applied multidimensional scaling (MDS) plot to evaluate gender outliers based on chromosome X data, that produced two separated clusters for male and female. We omitted 5 samples that do not belong to the distinct cluster. Furthermore, we applied median intensity plot for methylated and unmethylated intensity by using the minfi R package (3 samples below the 10.5 cutoff were excluded). All above led to exclusion of 8 samples.

Probes with a single nucleotide polymorphism in the single base extension site with a frequency of >5% were excluded (Chen et al. 2013), as were probes with non-optimal binding (non-mapping or mapping multiple times to either the normal or the bisulphite-converted genome), and the probed belonging to chrX and ChrY, resulting in the exclusion of 46,799 probes, leaving a total of 438,713 probes in the analysis.

Furthermore, we implemented "DASEN" recommended from wateRmelon package to do signal correction and normalization (Pidsley et al. 2013).

The Generation R Study

The Generation R Study is a population-based prospective cohort study from fetal life onwards in Rotterdam, the Netherlands, which has been previously described in detail (Jaddoe et al. 2012). Assessments in pregnant women consisted of physical examinations, fetal ultrasounds, biological samples, and questionnaires (Kruithof et al. 2014). All children were born between April 2002 and January 2006. The study has been approved by the Medical Ethical Committee of the Erasmus University Medical Centre and written consent was obtained for all participating mothers and children. For the current study, data was available for 809 Caucasian mothers and their children with information on NO_2 exposure during pregnancy and DNA-methylation at birth.

Generation R DNA methylation measurements

DNA was extracted from cord blood samples of 979 Caucasian children. Using the EZ-96 DNA Methylation kit (Shallow-well, Zymo Research Corporation, Irvine, USA), 500 ng DNA per sample underwent bisulfite conversion. Samples were transferred onto 96-well plates in a random order. Samples were processed with Illumina's Infinium HumanMethylation450 BeadChip (Illumina Inc., San Diego, USA). Quality control of analyzed samples was performed using standardized criteria. Samples were excluded due to sample call rate <99% (n=7) or poor bisulfite conversion (n=1). In addition, 2 samples were excluded because of a gender mismatch and 1 sample because of a retracted informed consent, leaving a total of 969 samples in the statistical analysis.

Probes with a single nucleotide polymorphism in the single base extension site with a frequency of >1% in the GoNLv4 reference panel were excluded, as were probes with non-optimal binding (non-mapping or mapping multiple times to either the normal or the

bisulphite-converted genome), resulting in the exclusion of 49,564 probes, leaving a total of 436,013 probes in the analysis.

Data were normalized with DASES normalization using a pipeline adapted from that developed by Touleimat and Tost (Touleimat and Tost 2012). DASES normalization includes background adjustment, between-array normalization applied to type I and type II probes separately, and dye bias correction applied to type I and type II probes separately. DASES is based on the DASEN method, but adds the dye bias correction, which is not included in DASEN (Pidsley et al. 2013). Beta-values were calculated for all CpG sites.

Air pollution exposure assessment in MeDALL, the Generation R Study and BAMSE EpiGene

The procedures for measurements and LUR modeling have been extensively described elsewhere (Pedersen et al. 2013; Van den Hooven et al. 2012). In short, 40 sampling sites (80 in the Netherlands/Belgium) for NO_2 and other agents were selected in each study area to characterize the spatial distribution of the cohort addresses, including regional background, urban background, and traffic sites. Measurements were performed at each site 3 times during 2 weeks in the cold, warm, and intermediate seasons, and the results were averaged to estimate the annual average. LUR models for NO_2 were developed based on measured annual average concentrations by using a range of Geographic Information System–derived predictor variables selected through a supervised stepwise procedure. Modeling was done locally at each center according to a common exposure assessment manual (http://www.escapeproject.eu/manuals/) following harmonized procedures regarding air pollutants measurements, development of land use regression models, and validation (Beelen et al. 2013). For the present analyses, total NO_2 exposure averaged throughout entire pregnancy was used. Data from routine monitoring stations were used to temporally adjust the LUR estimates to the periods corresponding to each individual pregnancy. The current exposure was estimated by assignment of modeled annual average NO_2 concentrations to the current addresses at the time when the blood samples were collected.

Children's Health Study (CHS)

CHS is a population-based prospective cohort study from age 6 onwards in Southern California, which has been described in detail elsewhere (McConnell et al. 2006). The study protocol was approved by the University of Southern California Institutional Review Board

and informed, written consent and assent were provided by the parents and children respectively.

A total of 5,341 children were recruited from schools within several Southern California communities, all of whom were born between 1995 and 1997 and are currently being followed until age 18. The cohort was established with the purpose of investigating the effects of air pollutants on respiratory health in children. Personal, parental, and socio-demographic characteristics were obtained by parent-completed questionnaire. Birth weight, gestational age, mode of delivery and other reproductive data were obtained from California birth records. The estimated date of conception was assigned using the birth date and gestational age, corrected for the average 2-week difference between the last menstrual period and conception. Ancestry (European, African, and Asian) was measured using ancestry informative markers SNPs and included as an additional covariate.

CHS DNA methylation measurements

Epigenome-wide DNA methylation was measured in 226 Hispanic and non-Hispanic white children, using DNA extracted from newborn bloodspots archived by the state of California. Laboratory personnel performing DNA methylation analysis were blinded to study subject information. DNA was extracted whole blood cells using the QiaAmp DNA blood kit (Qiagen Inc, Valencia, CA) and stored at -80 degrees Celcius. 700-1000ng of genomic DNA from each sample was treated with bisulfite using the EZ-96 DNA Methylation Kit™ (Zymo Research, Irvine, CA, USA), according to the manufacturer's recommended protocol and eluted in 18 ul. The results of the Infinium HumanMethylation450 BeadChip (HM450) were compiled for each locus as previously described and were reported as beta (β) values (Noushmehr et al. 2010).

CpG loci on the HM450 array were removed from analyses if they were on the X and Y chromosomes, or if they contained SNPs, deletions, repeats, or if they have more than 10% missing values. Data were processed in the methylumi package in R, after which a normal exponential background correction was applied to the raw intensities at the array level to reduce background noise (Triche et al. 2013). We then normalized each sample's methylation values to have the same quantiles to address sample to sample variability (Bolstad et al. 2003). β-values were calculated for all CpG sites.

CHS air pollution exposure assessment

The CHS air quality monitoring data (Peters et al. 1999a; Peters et al. 1999b) and the US EPA air Quality System were used to assign estimates of prenatal air pollution exposures for NO_2, based on a combination of residential history obtained from parents when subjects were 6-7 years old and birth address recorded on the birth certificate. In all but 34 cases, questionnaire-reported birth address from the residential history matched the birth address from the birth certificate. In the 34 cases where a mismatch was identified, the birth certificate address was used to assign air pollution exposure. Moreover, the birth address was representative of the mother's location throughout pregnancy in 88% of the subjects.

Addresses were geocoded using TeleAtlas Inc.'s Address Point Geocoding Services. Station-specific air quality data were spatially interpolated to each birth residence using inverse-distance-squared weighting (Hannam et al. 2013; Rivera-Gonzalez et al. 2015). The data from up to four air quality measurement stations were included in each interpolation with a maximum interpolation radius of 50 km. However, when a residence was located within 5 km of one or more stations with valid observations, the interpolation was based solely on the nearby values. Prenatal ambient air pollution concentrations were estimated for each subject's reported birth residence based on average monthly air pollutant exposure data. A leave one out evaluation of the spatial mapping method produced an r^2=0.73, for monthly NO_2 concentrations using data from California.

The Norwegian Mother and Child Cohort (MoBa)

MoBa is a prospective population-based pregnancy cohort study conducted by the Norwegian Institute of Public Health (Magnus et al. 2006; Ronningen et al. 2006). Participants were recruited from all over Norway from 1999-2008. The women consented to participation in 40.6% of the pregnancies. The cohort includes about 114,500 children, 95,200 mothers and 75,200 fathers. Blood samples were obtained from both parents during pregnancy and from mothers and children (umbilical cord) at birth. Data from MoBa was linked to the Medical Birth Registry of Norway (MBRN), and the current study used information from follow-up questionnaires at approximately 18-, and 30 - gestational weeks. DNA methylation and information on NO_2 exposure were available on 193 pregnancies. MoBa has obtained a licence from the Norwegian Data Inspectorate, and the current study was approved by The Regional Committee for Medical Research Ethics

MoBa DNA methylation measurements

Details on the DNA methylation and quality control can be found in (Joubert et al. 2016).

MoBa air pollution exposure assessment

Estimating air pollution exposure during pregnancy (including NO_2 measurements, LUR models development, and validation) was based on the methodology developed for the ESCAPE project (Beelen et al. 2013; Pedersen et al. 2013). LUR models for NO_2 levels were built for the studied areas in order to account for regional specifics. In the models we used air pollution measurements conducted in 2010 for Oslo and Akershus, and in 2011 for Bergen and Hordaland. Measurement campaigns included three rounds of approximately two weeks duration with NO_2 measurements (during winter, summer and an intermediate season) within a one year period. Measurement sites were selected to represent the range of residential exposure for each study area. For the analysis we included sites with no missing data, and no geocoding mismatches. LUR-modelled NO_2 yearly mean estimates for residential addresses at birth were temporally adjusted for each individual pregnancy using continuous routine monitoring station data. Daily NO_2 exposure estimates were averaged over the whole pregnancy.

Table S1. Characteristics of the included cohorts with available cord blood samples

	MeDALL cohorts						
**Cohort characteristics**	**BAMSE**	**EDEN**	**INMA**	**PIAMA**	**CHS**	**Generation R**	**MoBa**
Study design	Population based birth cohort.	Population based birth cohort, enrolled before 26 weeks of pregnancy	Population based birth cohort, enrolled at week 12 of pregnancy	Population based birth cohort (with mattress cover intervention and allergic/non-allergic parents)	Population-based children's cohort with retrospective collection of residential history, and archived newborn bloodspots	Population-based prospective birth cohort	Population based birth cohort
Age at enrolment	Newborns	Pregnant women		Pregnant women	5-6 years	Pregnant women	Pregnant women
Population source (area)	Stockholm, Sweden	Nancy and Poitiers, France	Sabadell, Spain	North, West and center of the Netherlands	Greater Los Angeles area	Rotterdam, the Netherlands	Norway
Enrolment period	1994-1996	2003-2006	2004-2007	1996-1997	2002	2002-2006	1999-2008
Cohort recruitment	Community population register	Prenatal Healthcare	Prenatal healthcare	Prenatal healthcare	Community schools	Prenatal healthcare	Prenatal healthcare
Total number of recruited children	4,089	2,002	638	3,963	5341	9,901	
Follow-up time points (year of life)	1,2,4,8	Birth, 4 and 8 months, 1, 2, 3, 4, 5-6, 8 years	Birth, 1, 2, 4, 7, 9	1,2,3,4,5,6,7,8, 11,14	6-18		Birth, 0.5, 1.5, 3, 5, 7
**Traffic air pollution**							
Estimation model	LUR	LUR	LUR	LUR	Dispersion	LUR	LUR
Time of measurements	2009	2002, 2005	2010	2009	1995-1996	2002-2006	2010, 2011
**Sample selection**							
Criteria for selection of sample	4yr – 8yr paired DNA samples. Asthma case-control	Birth-5yr paired DNA samples.	Birth-4yr paired DNA samples. Asthma case-control	4yr – 8yr paired DNA samples. Asthma case-control	Randomly selected from within subjects that matched to CA birth records and had a bloodspot	European ancestry	

Methylation measurement platform	Illumina 450K assay	Illumina 450K assay	Illumina 450K assay	Illumina 450K assay	Illumina 450K assay	Illumina 450K assay	Illumina 450K assay
Number of CpGs available per cohort	439,306	439,306	439,306	439,306	368,386	436,013	473,731
Ethnicity	Caucasian	Caucasian	Caucasian	Caucasian	Hispanic white and non-Hispanic white	Caucasian	Caucasian

A) cg12283362 (*LONP1*)

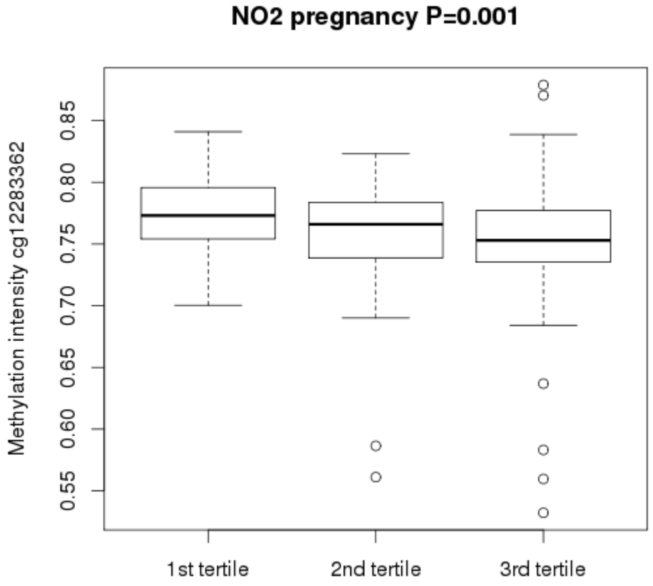

B) cg24172570 (3.8 kbp upstream of *HIBADH*)

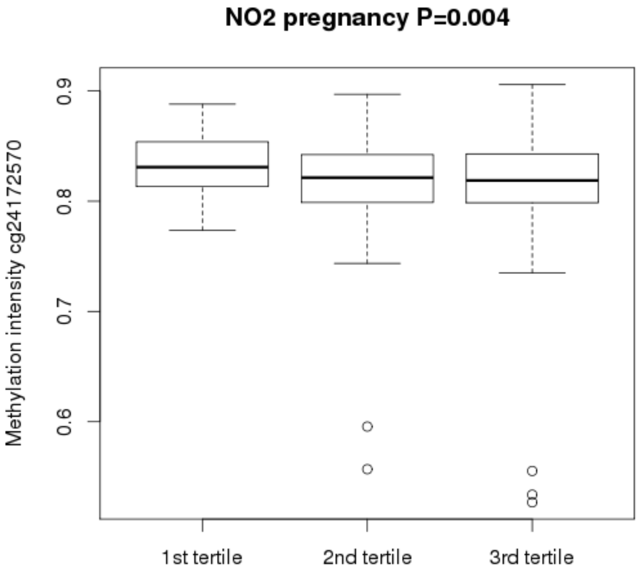

C) cg08973675 (*SLC25A28*)

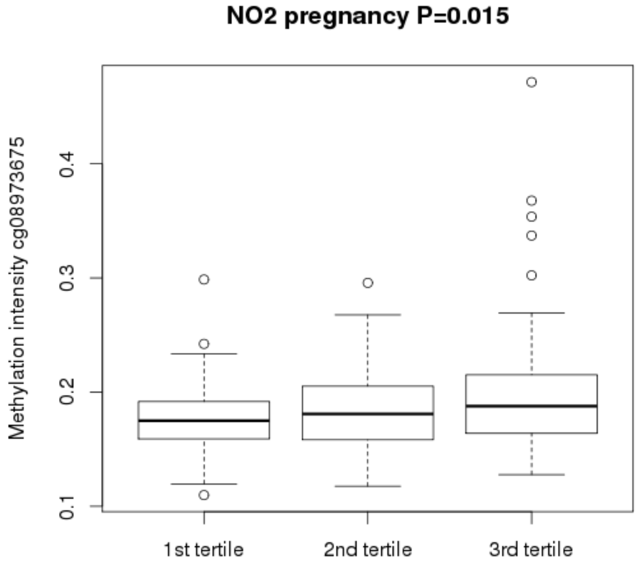

Figure S1. Plot of the cumulative distribution function for methylation intensities for the three top-ranked CpG sites: cg12283362 (*LONP1*), cg24172570 (3.8 kbp upstream of *HIBADH*), and cg08973675 (*SLC25A28*) by the cohort-specific tertiles of prenatal NO_2 exposure (in the INMA cohort, 1st tertile <39.9 µg/m^3; 2nd tertile 39.9-47.6 µg/m^3; 3rd tertile >47.6 µg/m^3; in the EDEN cohort, 1st tertile <12.7 µg/m^3; 2nd tertile 12.7-17.6 µg/m^3; 3rd tertile >17.6 µg/m^3) demonstrates a statistically significant dose-response effect of exposure in the INMA and EDEN cohorts. The p-values are derived from the Jonckheere-Terpstra trend test, calculated using the SAGx package in R, version 2.14.0.

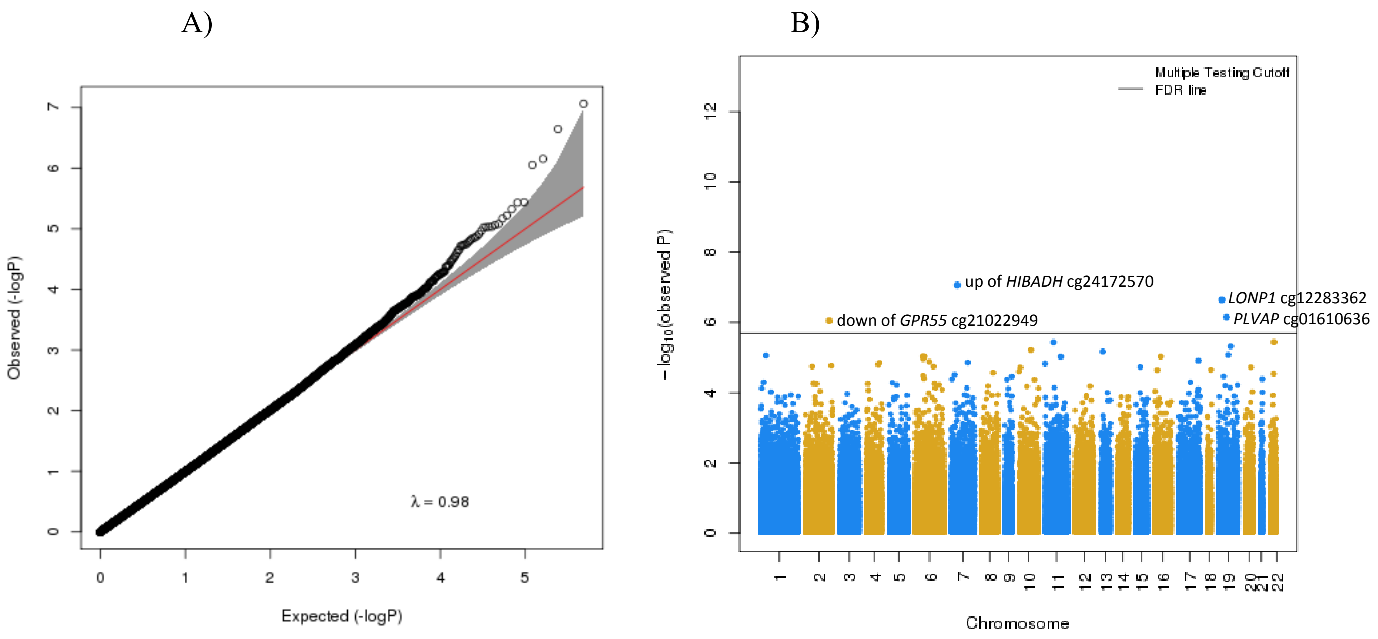

Figure S2. Quantile-quantile plot (A) and Manhattan plot (B) for epigenome-wide meta-analysis of the association between NO_2 exposure during pregnancy and cord blood DNA methylation, additionally adjusted for cell type composition (n=1,508). Four CpGs were considered statistically significant using FDR correction (solid horizontal line): cg24172570 3.8 kbp upstream of *HIBADH*, cg12283362 in *LONP1*, cg01610636 in *PLVAP*, and cg21022949 19.7 kbp downstream of *GPR55*.

Table S2. Top 25 CpGs from the epigenome-wide meta-analysis of the association between NO_2 exposure during pregnancy and cord blood DNA methylation additionally adjusted for cell composition (n=1,508 newborns from MeDALL, Generation R, CHS and MoBa cohorts).

Chr	Position (build 37)	CpG	Mapped gene	Gene group	Coef	SE	P-value	Direction
7	27561178	cg24172570	*HIBADH** FDR		-0.004	8.00E-04	8.65E-08	--?-
19	5709149	cg12283362	*LONP1* FDR	Body	-0.007	1.30E-03	2.27E-07	-??-
19	17463255	cg01610636	*PLVAP* FDR	Body	-0.005	9.00E-04	7.03E-07	----
2	231809697	cg21022949	*GPR55** FDR		0.001	2.00E-04	8.86E-07	++++
22	40355732	cg17988310	*GRAP2*	Body	0.004	8.00E-04	3.65E-06	++++
11	47400146	cg03565868	*SPI1*	TSS200	0.005	1.10E-03	3.71E-06	+++-
19	39884218	cg14651844	*MED29*	Body	0.002	5.00E-04	4.75E-06	+-++
10	101380289	cg08973675	*SLC25A28*	TSS200	0.005	1.10E-03	6.05E-06	+++-
13	32524761	cg00648883	*EEF1DP3*	Body	0.006	1.30E-03	6.77E-06	++++
19	30155866	cg05512099	*PLEKHF1*	TSS1500	-0.007	1.60E-03	8.36E-06	-+?-
1	9675560	cg25590444	*TMEM201**		0.003	6.00E-04	8.67E-06	+-++
6	31382102	cg26504614	*MICA*	Body	-0.005	1.10E-03	9.25E-06	-?--
6	30524763	cg03860665	*PRR3*	5'UTR; 1stExon	0.002	4.00E-04	9.27E-06	++++
16	27325254	cg06641959	*IL4R*	5'UTR;1stExon	0.002	4.00E-04	9.38E-06	++++
11	74871202	cg12537437	*SLCO2B1*	Body;5'UTR	-0.003	7.00E-04	9.47E-06	---+
6	30689865	cg25742745	*TUBB*	Body	0.004	1.00E-03	1.09E-05	+??+
17	78926091	cg21831512	*RPTOR*	Body	0.004	8.00E-04	1.22E-05	+--+
6	33359817	cg04757012	*KIFC1*	Body	0.001	3.00E-04	1.33E-05	++++
7	117824040	cg08301459	*NAA38*	TSS200	0.002	3.00E-04	1.39E-05	++?+
4	154386136	cg21908828	*KIAA0922*	TSS1500	0.004	1.00E-03	1.42E-05	++++
11	369155	cg19787465	*B4GALNT4*	TSS1500	0.004	8.00E-04	1.49E-05	+++-
4	139940407	cg04702527	*CCRN4L*	Body	-0.005	1.20E-03	1.58E-05	-+?-
2	239984105	cg09155776	*HDAC4*	Body	-0.010	2.30E-03	1.69E-05	----
2	60698937	cg04588436	*BCL11A*	Body	-0.003	8.00E-04	1.80E-05	-??-
6	56114591	cg11241549			0.007	1.50E-03	1.81E-05	+??+

Shown are top 25 CpGs ordered by p-value; All results presented per 10 μg/m^3 increase in prenatal NO_2 exposure. Column headers: CHR: chromosome; Position: Chromosomal position based on NCBI human reference genome assembly Build 37. Mapped Gene: UCSC annotated gene; Gene Group: UCSC gene region feature category; regression coefficient; SE: standard error for regression coefficient; Direction: Direction of effect across cohorts included in the statistical model (MeDALL, Generation R, CHS and MoBa): NO_2 exposure during pregnancy associated with increased (+) or decreased (-) methylation, or missing (?) result. Genome-wide significance threshold (FDR p<0.05).

*cg24172570 is located 3.8 kbp upstream of *HIBADH*; cg21022949 – 19.7 kbp downstream of *GPR55*; cg25590444 - 0,6 kbp downstream of *TMEM201*.

** Data on methylation of cg12283362 was available in 473 individuals, cg24172570 - in 1282 individuals.

Table S3. Top 25 CpGs from the analysis of the association between NO_2 exposure during pregnancy and cord blood DNA methylation without adjustment for cell composition (upper part) and with adjustment for cell composition according to Bakulski (lower part) (n=280 newborns from the MeDALL study).

Unadjusted for celltype							
**NO_2 at pregnancy → Methylation at birth (EDEN+INMA n=280)**							
**Chr**	**Position (build 37)**	**CpG**	**Mapped gene**	**Gene group**	**Coef**	**SE**	**P-value**
17	43394547	cg03787849	*MAP3K14* [FDR]	Island	0.002	4.09E-04	7.69E-08
19	5709149	cg12283362	*LONP1* [FDR]	S_Shore	-0.007	1.42E-03	3.62E-07
11	62439187	cg26435734	*C11orf83;C11orf48* [FDR]	Island	0.002	4.03E-04	5.72E-07
12	121124951	cg26413987	*MLEC* [FDR]	Island	0.002	3.34E-04	7.24E-07
1	24127146	cg13180787	*GALE* [FDR]	Island	0.004	7.56E-04	1.15E-06
19	14201991	cg17688733	*SAMD1* [FDR]	OpenSea	0.003	5.89E-04	1.37E-06
2	122494609	cg04002021	*MKI67IP* [FDR]	S_Shore	0.003	6.14E-04	1.46E-06
14	74416831	cg19496328	*FAM161B;COQ6* [FDR]	N_Shore	0.002	5.21E-04	1.67E-06
2	25896320	cg20347626	*DTNB* [FDR]	Island	0.002	3.65E-04	2.10E-06
19	4066280	cg06403289	*ZBTB7A* [FDR]	Island	0.005	9.65E-04	2.16E-06
6	33290736	cg07689821	*DAXX*	Island	0.005	9.54E-04	2.18E-06
8	65711658	cg01510388	*CYP7B1*	Island	-0.011	2.36E-03	2.72E-06
6	31869521	cg11801452	*ZBTB12*	Island	0.003	6.09E-04	2.85E-06
7	27561178	cg24172570	*HIBADH**	OpenSea	-0.005	1.05E-03	3.35E-06
10	101380289	cg08973675	*SLC25A28*	Island	0.007	1.48E-03	3.47E-06
3	52001995	cg07110217	*PCBP4*	OpenSea	0.002	5.18E-04	3.61E-06
11	118307669	cg18705039	*MLL*	Island	0.009	1.92E-03	4.13E-06
6	28351351	cg15964468	*ZSCAN12*	Island	0.009	2.04E-03	4.31E-06
6	30899568	cg26672776	*SFTA2*	OpenSea	-0.014	3.13E-03	4.38E-06
2	110873741	cg09113530	*MALL*	S_Shore	-0.007	1.52E-03	4.62E-06
4	95679705	cg09156233	*BMPR1B*	Island	-0.001	1.87E-04	4.74E-06
11	1968310	cg18482326	*MRPL23*	N_Shore	0.005	1.18E-03	5.44E-06
19	39897430	cg07051257	*ZFP36*	Island	0.006	1.27E-03	5.68E-06
12	7070317	cg22260508	*PTPN6*	OpenSea	0.004	8.60E-04	6.17E-06
17	78851213	cg08314949	*RPTOR*	S_Shore	0.016	3.46E-03	6.29E-06
**Adjusted for celltype according to Bakulski**							
**NO_2 at pregnancy → Methylation at birth (EDEN+INMA n=280)**							
**Chr**	**Position (build 37)**	**CpG**	**Mapped gene**	**Gene group**	**Coef**	**SE**	**P-value**
19	5709149	cg12283362	*LONP1* [FDR]	S_Shore	-0.007	1.28E-03	1.37E-07
2	122494609	cg04002021	*MKI67IP* [FDR]	S_Shore	0.003	5.40E-04	1.71E-07
6	43244304	cg06241901	*TTBK1* [FDR]	Island	-0.008	1.68E-03	6.08E-07
17	43394547	cg03787849	*MAP3K14* [FDR]	Island	0.002	4.49E-04	6.08E-07
17	42989137	cg20911989	*GFAP* [FDR]	Island	-0.009	1.72E-03	7.31E-07
12	121124951	cg26413987	*MLEC* [FDR]	Island	0.002	3.29E-04	8.23E-07
17	77768687	cg04986373	*CBX8* [FDR]	N_Shore	0.006	1.31E-03	9.56E-07
8	65711658	cg01510388	*CYP7B1* [FDR]	Island	-0.011	2.24E-03	1.13E-06
14	74416831	cg19496328	*FAM161B;COQ6* [FDR]	N_Shore	0.002	5.01E-04	1.50E-06

11	118307669	cg18705039	*MLL* FDR	Island	0.009	1.83E-03	1.54E-06
19	4066280	cg06403289	*ZBTB7A* FDR	Island	0.005	9.70E-04	1.57E-06
2	241498221	cg24958325	*ANKMY1;DUSP28* FDR	N_Shore	0.004	7.30E-04	1.58E-06
6	30899568	cg26672776	*SFTA2* FDR	OpenSea	-0.014	2.98E-03	1.72E-06
3	52443970	cg10956904	*BAP1;PHF7* FDR	Island	0.003	5.35E-04	1.89E-06
16	75021994	cg06383207		S_Shelf	-0.007	1.56E-03	2.41E-06
20	3030247	cg25472862		S_Shelf	0.003	7.39E-04	2.46E-06
10	101380289	cg08973675	*SLC25A28*	Island	0.007	1.45E-03	2.71E-06
19	14201991	cg17688733	*SAMD1*	OpenSea	0.003	5.60E-04	2.88E-06
6	33393786	cg21726235	*SYNGAP1*	Island	0.004	8.75E-04	2.95E-06
3	52001995	cg07110217	*PCBP4*	OpenSea	0.002	5.32E-04	3.44E-06
8	48099615	cg03271173		N_Shore	-0.003	5.87E-04	3.88E-06
1	1821981	cg03716942	*GNB1*	Island	0.002	4.87E-04	4.56E-06
16	30106682	cg26709300	*YPEL3*	N_Shore	0.006	1.41E-03	5.11E-06
19	17463255	cg01610636	*PLVAP*	S_Shore	-0.005	1.13E-03	5.16E-06
11	62439187	cg26435734	*C11orf83;C11orf48*	Island	0.002	4.22E-04	5.30E-06

Estimates are presented per 10 µg/m^3 increase in NO_2 exposure. Column headers: CHR: chromosome; Position: Chromosomal position based on NCBI human reference genome assembly Build 37. Mapped Gene: UCSC annotated gene; Gene Group: UCSC gene region feature category; regression coefficient; SE: standard error for regression coefficient;
*cg24172570 is located 3.8 kbp upstream of *HIBADH*.

Table S4. Look-up in older children for top 25 findings for prenatal NO_2 exposure in relation to methylation in meta-analysis of newborns.

Meta-analysis of cord blood results (MeDALL + Generation R + CHS + MoBa)									Replication in 4-year-old children of the MeDALL cohorts						Replication in 8-year-old children of the MeDALL + EpiGene cohorts (meta-analysis)					
NO_2 pregnancy → Methylation at birth (n=1,508)									NO_2 at pregnancy→ Methylation at 4 yrs (n=733)			NO_2 at 4 yrs → Methylation at 4 yrs (n=689)			NO_2 at pregnancy → Methylation at 8 yrs (n=786)			NO_2 at 8 yrs → Methylation at 8 yrs (n=829)		
**Chr**	**Position (build 37)**	**CpG**	**Mapped gene**	**Gene group**	**Coef**	**SE**	**P-value**	**Direction**	**Coef**	**SE**	**P-value**	**Coef**	**SE**	**P-value**	**Coef**	**SE**	**P-value**	**Coef**	**SE**	**P-value**
19	5709149	cg12283362	*LONP1* [FDR]	Body	-0.007	1.40E-03	1.78E-07	-??-**	0.002	0.0012	0.21	0.001	0.0012	0.37	0.000	0.0013	0.95	0.002	0.0019	0.24
7	27561178	cg24172570	*HIBADH** [FDR]		-0.004	8.00E-04	3.01E-07	--?-**	0.001	0.0009	0.16	0.002	0.0012	0.19	0.001	0.0015	0.69	0.003	0.0020	0.18
10	101380289	cg08973675	*SLC25A28* [FDR]	TSS200	0.005	1.10E-03	2.20E-06	++++	0.002	0.0011	**0.03**	0.001	0.0012	0.47	0.003	0.0012	**0.04**	0.003	0.0019	0.11
22	40355732	cg17988310	*GRAP2*	Body	0.004	9.00E-04	5.25E-06	++++	0.001	0.0014	0.43	0.002	0.0018	0.24	-0.001	0.0017	0.69	0.003	0.0026	0.22
20	61427684	cg14582546	*C20orf20*	TSS200	0.005	1.10E-03	5.50E-06	++++	-0.001	0.0013	0.30	-0.001	0.0011	0.30	0.001	0.0010	0.44	0.001	0.0016	0.39
22	39323510	cg12276768	*APOBEC3A**		0.003	6.00E-04	5.60E-06	++++	0.000	0.0009	0.77	0.001	0.0009	0.22	0.002	0.0010	**0.04**	0.002	0.0014	0.16
6	30688588	cg21660604	*TUBB*	Body	0.002	3.00E-04	8.36E-06	++++	0.000	0.0004	0.45	0.000	0.0004	0.86	0.000	0.0004	0.84	0.001	0.0007	0.06
5	77284206	cg26815688	*AP3B1**		-0.002	5.00E-04	9.03E-06	----	0.000	0.0006	0.41	0.001	0.0006	0.16	0.001	0.0006	0.27	-0.001	0.0009	0.40
6	30524763	cg03860665	*PRR3;GNL1*	5'UTR;1stExon	0.002	5.00E-04	9.17E-06	++++	0.001	0.0007	0.29	0.000	0.0007	0.68	0.000	0.0009	0.63	0.001	0.0011	0.57
7	117824040	cg08301459	*NAA38*	TSS200	0.002	3.00E-04	9.58E-06	++?+	0.001	0.0005	0.12	0.001	0.0006	0.07	0.000	0.0005	0.75	0.000	0.0007	0.62
6	33359817	cg04757012	*KIFC1*	Body	0.001	3.00E-04	1.01E-05	++++	0.000	0.0004	0.76	0.000	0.0005	0.82	0.000	0.0004	0.56	0.000	0.0006	0.86
11	74871202	cg12537437	*SLCO2B1*	Body;5'UTR	-0.004	8.00E-04	1.02E-05	---+	-0.001	0.0007	0.34	0.000	0.0007	0.86	0.001	0.0009	0.40	0.001	0.0013	0.47
1	35226135	cg01828548	*GJB4*	5'UTR	-0.005	1.10E-03	1.08E-05	---+	0.001	0.0012	0.22	0.001	0.0015	0.40	-0.004	0.0015	**0.01**	-0.006	0.0023	**0.01**
21	46032086	cg26386968	*C21orf29; KRTAP10-8*	Body;1stExon	-0.007	1.50E-03	1.15E-05	--?-	-0.003	0.0011	**0.02**	-0.002	0.0013	0.11	-0.002	0.0014	0.11	-0.002	0.0022	0.27
9	139607421	cg12657416	*FAM69B*	Body	0.103	2.36E-02	1.22E-05	?+?+	NA	NA	NA	NA	NA	NA	NA	NA	NA	NA	NA	NA
11	34460856	cg03728580	*CAT*	Body	0.003	7.00E-04	1.43E-05	++++	0.000	0.0010	1.00	0.000	0.0012	0.85	0.000	0.0010	0.97	0.001	0.0016	0.68
2	231809697	cg21022949	*GPR55**		0.001	2.00E-04	1.51E-05	++++	0.000	0.0003	0.77	0.000	0.0004	0.68	0.001	0.0004	**0.001**	0.000	0.0006	0.89
2	98409069	cg06840305	*TMEM131*	Body	-0.002	4.00E-04	1.51E-05	----	0.000	0.0006	0.70	0.001	0.0006	0.10	0.000	0.0008	0.84	0.002	0.0011	**0.04**
12	120967065	cg11075121	*COQ5*	TSS200	0.002	4.00E-04	1.66E-05	++++	0.000	0.0006	0.48	0.001	0.0007	0.40	0.001	0.0006	0.06	0.002	0.0009	**0.03**
8	48099615	cg03271173	*IGLV8OR8-1**		-0.003	6.00E-04	1.70E-05	--+-	-0.001	0.0005	0.33	0.000	0.0007	0.89	0.000	0.0007	0.58	0.000	0.0010	0.75
8	110346503	cg25407888	*ENY2;NUDCD1*	TSS200	0.003	6.00E-04	1.98E-05	++++	0.000	0.0009	0.70	0.000	0.0009	0.58	0.000	0.0009	0.74	0.000	0.0011	0.73
21	45753677	cg24316255	*C21orf2*	Body	-0.003	6.00E-04	2.00E-05	---+	-0.001	0.0007	0.14	0.000	0.0009	1.00	-0.001	0.0009	0.12	-0.001	0.0012	0.57
17	78851213	cg08314949	*RPTOR*	Body;Body	0.013	3.10E-03	2.06E-05	+-?+	0.015	0.0039	**0.0001**	0.010	0.0047	**0.04**	-0.006	0.0053	0.23	0.003	0.0072	0.66
15	59063272	cg01889112	*FAM63B*	TSS200;TSS200	0.002	4.00E-04	2.29E-05	++++	0.000	0.0006	0.89	0.001	0.0007	0.40	0.000	0.0006	0.75	0.001	0.0009	0.45
6	31382102	cg26504614	*MICA*	Body	-0.005	1.10E-03	2.59E-05	-?--	0.000	0.0010	0.65	0.000	0.0010	0.97	-0.003	0.0010	**0.001**	-0.001	0.0015	0.61

Shown are top 25 CpGs from the discovery meta-analysis ordered by p-value; Estimates are presented per 10 µg/m^3 increase in NO_2 exposure.Chr: chromosome; Direction: Direction of effect across cohorts included in the meta-analysis (MeDALL, Generation R, CHS and MoBa): NO_2 exposure during pregnancy associated with increased (+) or decreased (-) methylation, or missing (?) result; NA=not available.

* cg24172570 is located 3.8 kbp upstream of *HIBADH, cg12276768 – 25.2* kbp upstream of *APOBEC3A,* cg26815688 - 21.1 kbp upstream of *AP3B1,* cg21022949 – 19.7 kbp downstream of *GPR55*, cg03271173 - *14.5* kbp upstream of *IGLV8OR8-1.*

** Data on methylation of cg12283362 was available in 473 individuals, cg24172570 - in 1282 individuals.

Table S5. Look-up in 4-year-old children with paired cord blood and 4-year-old samples for top 25 findings for prenatal NO_2 exposure in relation to methylation in meta-analysis of newborns.

Meta-analysis of cord blood results (MeDALL + Generation R + CHS + MoBa)									Replication in 4-year-old children of the MeDALL cohorts		
NO_2 pregnancy → Methylation at birth (n=1,508)									NO_2 pregnancy→ Methylation at 4 yrs INMA+EDEN (n=277)		
**Chr**	**Position (build 37)**	**CpG**	**Mapped gene**	**Gene group**	**Coef**	**SE**	**P-value**	**Direction**	**Coef**	**SE**	**P-value**
19	5709149	cg12283362	*LONP1* [FDR]	Body	-0.007	1.40E-03	1.78E-07	-??-**	0.001	1.58E-03	0.42
7	27561178	cg24172570	*HIBADH** [FDR]		-0.004	8.00E-04	3.01E-07	--?-**	0.001	1.28E-03	0.54
10	101380289	cg08973675	*SLC25A28* [FDR]	TSS200	0.005	1.10E-03	2.20E-06	++++	0.004	1.46E-03	**0.005**
22	40355732	cg17988310	*GRAP2*	Body	0.004	9.00E-04	5.25E-06	++++	0.005	2.03E-03	**0.02**
20	61427684	cg14582546	*C20orf20*	TSS200	0.005	1.10E-03	5.50E-06	++++	0.001	2.02E-03	0.61
22	39323510	cg12276768	*APOBEC3A**		0.003	6.00E-04	5.60E-06	++++	0.001	9.76E-04	0.45
6	30688588	cg21660604	*TUBB*	Body	0.002	3.00E-04	8.36E-06	++++	0.000	5.56E-04	0.55
5	77284206	cg26815688	*AP3B1**		-0.002	5.00E-04	9.03E-06	----	0.000	8.77E-04	0.66
6	30524763	cg03860665	*PRR3;GNL1*	5'UTR;1stExon	0.002	5.00E-04	9.17E-06	++++	0.001	8.84E-04	0.37
7	117824040	cg08301459	*NAA38*	TSS200	0.002	3.00E-04	9.58E-06	++?+	0.002	6.55E-04	**0.001**
6	33359817	cg04757012	*KIFC1*	Body	0.001	3.00E-04	1.01E-05	++++	0.001	5.24E-04	0.05
11	74871202	cg12537437	*SLCO2B1*	Body;5'UTR	-0.004	8.00E-04	1.02E-05	---+	-0.001	9.14E-04	0.11
1	35226135	cg01828548	*GJB4*	5'UTR	-0.005	1.10E-03	1.08E-05	---+	-0.002	1.57E-03	0.23
21	46032086	cg26386968	*C21orf29;KRTAP10-8*	Body;1stExon	-0.007	1.50E-03	1.15E-05	--?-	-0.006	1.53E-03	**0.00005**
9	139607421	cg12657416	*FAM69B*	Body	0.103	2.36E-02	1.22E-05	?+?+	NA	NA	NA
11	34460856	cg03728580	*CAT*	Body	0.003	7.00E-04	1.43E-05	++++	0.000	1.41E-03	0.77
2	231809697	cg21022949	*GPR55**		0.001	2.00E-04	1.51E-05	++++	0.000	4.22E-04	0.95
2	98409069	cg06840305	*TMEM131*	Body	-0.002	4.00E-04	1.51E-05	----	-0.001	9.01E-04	0.33
12	120967065	cg11075121	*COQ5*	TSS200	0.002	4.00E-04	1.66E-05	++++	0.001	8.22E-04	0.14
8	48099615	cg03271173	*IGLV8OR8-1**		-0.003	6.00E-04	1.70E-05	--+-	-0.001	8.26E-04	0.10
8	110346503	cg25407888	*ENY2;NUDCD1*	TSS200	0.003	6.00E-04	1.98E-05	++++	0.000	1.40E-03	0.81
21	45753677	cg24316255	*C21orf2*	Body	-0.003	6.00E-04	2.00E-05	---+	-0.002	1.07E-03	0.15
17	78851213	cg08314949	*RPTOR*	Body;Body	0.013	3.10E-03	2.06E-05	+-?+	0.017	4.41E-03	**0.0002**
15	59063272	cg01889112	*FAM63B*	TSS200;TSS200	0.002	4.00E-04	2.29E-05	++++	0.001	8.21E-04	0.19
6	31382102	cg26504614	*MICA*	Body	-0.005	1.10E-03	2.59E-05	-?--	0.000	1.23E-03	0.81

Shown are top 25 CpGs from the discovery meta-analysis ordered by p-value; Estimates are presented per 10 μg/m^3 increase in NO_2 exposure. Column headers: CHR: chromosome; Position: Chromosomal position based on NCBI human reference genome assembly Build 37. Mapped Gene: UCSC annotated gene; Gene Group: UCSC gene region feature category; regression coefficient; SE: standard error for regression coefficient; Direction: Direction of effect across cohorts included in the statistical model (MeDALL, Generation R, CHS and MoBa): NO_2 exposure during pregnancy associated with increased (+) or decreased (-) methylation, or missing (?) result. NA=not available

* cg24172570 is located 3.8 kbp upstream of *HIBADH, cg12276768 – 25.2* kbp upstream of *APOBEC3A,* cg26815688 - 21.1 kbp upstream of *AP3B1,* cg21022949 – 19.7 kbp downstream of *GPR55*, cg03271173 - *14.5* kbp upstream of *IGLV8OR8-1*.

** Data on methylation of cg12283362 was available in 473 individuals, cg24172570 - in 1282 individuals.

Table S6. Nominally significant CpGs within oxidative stress genes extracted from the epigenome-wide meta-analysis of the association between prenatal NO_2 exposure and newborn cord blood DNA methylation (n=1,508 newborns from MeDALL, Generation R, CHS and MoBa cohorts).

Chr	Position (build 37)	CpG	Mapped gene	Gene group	Coef	SE	P-value	Direction
11	34460856	cg03728580	*CAT* [FDR]	Body	0.003	0.001	**0.00001**	++++
11	34461028	cg17034036	*CAT* [FDR]	Body	0.002	0.001	**0.0001**	++++
2	1482597	cg01385533	*TPO* [FDR]	Body	-0.003	0.001	**0.0004**	-?--
1	226023590	cg05935800	*EPHX1*	Body	-0.002	0.001	**0.002**	----
20	33539306	cg13607138	*GSS*	Body	-0.003	0.001	**0.003**	--?-
8	107642385	cg17526936	*OXR1*	Body	-0.002	0.001	**0.004**	--?-
2	1544120	cg19407717	*TPO*	Body	-0.002	0.001	**0.004**	----
2	1479523	cg13703866	*TPO*	Body	-0.001	0.000	0.005	----
11	34460336	cg07768201	*CAT*	TSS200	0.003	0.001	0.006	++++
1	226012507	cg03337430	*EPHX1*	TSS1500;5'UTR	0.001	0.000	0.006	+-++
2	1416855	cg08946720	*TPO*	TSS1500	-0.002	0.001	0.006	--?+
2	1743009	cg01821226	*PXDN*	Body	-0.001	0.001	0.007	----
2	1654326	cg08380973	*PXDN*	Body	-0.003	0.001	0.009	----
2	1518383	cg24215279	*TPO*	Body	0.001	0.000	0.011	+-+-
6	160113813	cg14515483	*SOD2*	Body	0.001	0.000	0.012	++++
2	1652518	cg26063629	*PXDN*	Body	-0.002	0.001	0.012	---+
11	34460351	cg03720043	*CAT*	TSS200	0.003	0.001	0.012	++++
11	67351273	cg26250609	*GSTP1*	1stExon;5'UTR	0.002	0.001	0.013	++++
17	26120702	cg01396112	*NOS2*	Body	0.000	0.000	0.016	---+
2	1488516	cg02892893	*TPO*	Body	-0.001	0.001	0.018	--?-
15	45406362	cg10778736	*DUOX2*	TSS200	0.002	0.001	0.018	++++
15	45405370	cg07821960	*DUOX2*	Body;TSS1500	0.001	0.000	0.018	++++
17	26084080	cg15088880	*NOS2*	3'UTR	0.005	0.002	0.019	??+-
8	52618305	ch.8.1157478F	*PXDNL*	Body	0.004	0.002	0.020	++?+
2	1425205	cg06972972	*TPO*	Body	-0.001	0.000	0.021	----
2	201450731	cg12627583	*AOX1*	1stExon;5'UTR	0.001	0.001	0.021	+++-
7	2289888	cg13748354	*NUDT1*	Body	0.038	0.016	0.022	?+?+
2	1478219	cg25013910	*TPO*	Body	-0.004	0.002	0.022	--?-
11	67350499	cg05244766	*GSTP1*	TSS1500	-0.002	0.001	0.024	-?-+
3	38207809	cg15789250	*OXSR1*	Body	0.005	0.002	0.025	++++
2	201450690	cg02144933	*AOX1*	TSS200	0.001	0.000	0.029	++++
16	69760301	cg06790860	*NQO1*	Body	0.001	0.000	0.032	+-++
2	1668758	cg12624031	*PXDN*	Body	-0.001	0.001	0.034	----
17	56274480	cg08105265	*EPX*	Body	0.002	0.001	0.034	+?++
8	52721657	cg02090805	*PXDNL*	Body	0.001	0.001	0.035	+++-
8	107738435	cg12114888	*OXR1*	Body	0.005	0.002	0.037	-+++
2	1682068	cg09996777	*PXDN*	Body	-0.003	0.001	0.040	--?-
2	1493713	cg06173919	*TPO*	Body	-0.002	0.001	0.041	--?+

1	225996810	cg21826272	*EPHX1*	TSS1500	-0.002	0.001	0.043	--?-
7	2281541	cg04305677	*NUDT1*	TSS1500	0.000	0.000	0.044	--++
11	67350491	cg08925882	*GSTP1*	TSS1500	-0.004	0.002	0.045	??--
2	178128234	cg16842060	*NFE2L2*	Body;1stExon;5'UTR	0.000	0.000	0.047	+-++
7	2284600	cg03663120	*NUDT1*	Body	0.007	0.004	0.048	++?+
1	53068579	cg23272399	*GPX7*	Body	-0.001	0.001	0.049	----
6	160114681	cg04311230	*SOD2*	TSS1500	0.000	0.000	0.049	-+++

Shown here are the 45 nominally significant ($p<0.05$) CpGs ordered by p-value. Three CpGs were statistically significant using genome-wide significance threshold (FDR $p<0.05$). Results presented per 10 $\mu g/m^3$ increase in prenatal NO_2 exposure.

Column headers: CHR=chromosome; Position=Chromosomal position based on NCBI human reference genome assembly Build 37. Mapped Gene=UCSC annotated gene; Gene Group=UCSC gene region feature category; regression coefficient; SE=standard error for regression coefficient; Direction=Direction of effect across cohorts included in the statistical model (MeDALL, Generation R, CHS and MoBa): NO_2 exposure during pregnancy associated with increased (+) or decreased (-) methylation, or missing (?) result.

Table S7. All available CpGs mapped to *CAT* gene from the epigenome-wide meta-analysis of the association between NO_2 exposure during pregnancy and newborn cord blood DNA methylation (N=1,508 newborns from MeDALL, Generation R, CHS and MoBa cohorts).

Chr	Position (build 37)	CpG	Mapped gene	Gene group	Coef	SE	P-value	Direction
11	34460856	cg03728580	*CAT*	Island	0.003	7.00E-04	**0.00001**	++++
11	34461028	cg17034036	*CAT*	S_Shore	0.002	6.00E-04	**0.0001**	++++
11	34460336	cg07768201	*CAT*	Island	0.003	1.10E-03	**0.01**	++++
11	34460351	cg03720043	*CAT*	Island	0.003	1.00E-03	**0.01**	++++
11	34460516	cg06908474	*CAT*	Island	0.002	1.00E-03	0.07	++++
11	34460386	cg02109652	*CAT*	Island	0.002	1.00E-03	0.12	-+++
11	34460107	cg20234170	*CAT*	N_Shore	0.001	8.00E-04	0.19	++-+
11	34460557	cg01847719	*CAT*	Island	0.002	1.50E-03	0.22	++++
11	34460182	cg22159421	*CAT*	Island	0.001	6.00E-04	0.23	+-++
11	34460318	cg06027906	*CAT*	Island	0.000	3.00E-04	0.24	++++
11	34460172	cg24099074	*CAT*	Island	0.000	2.00E-04	0.25	++++
11	34460298	cg20731136	*CAT*	Island	0.001	4.00E-04	0.26	+-++
11	34464477	cg09106728	*CAT*	S_Shelf	-0.001	1.60E-03	0.48	--?+
11	34492984	cg17098995	*CAT*	OpenSea	0.000	3.00E-04	0.82	--+-
11	34460789	cg14316565	*CAT*	Island	0.000	6.00E-04	0.96	+-++

Shown are all CpGs within *CAT* gene ordered by p-value; All results presented per 10 µg/m^3 increase in prenatal NO_2 exposure. Column headers: CHR: chromosome; Mapped Gene: UCSC annotated gene; Gene Group: UCSC gene region feature category; Coef: regression coefficient; Direction: Direction of effect across cohorts included in the statistical model (MeDALL, Generation R, CHS and MoBa): NO_2 exposure during pregnancy associated with increased (+) or decreased (-) methylation, or missing (?) result.

Table S8. All available CpGs mapped to *TPO* gene from the epigenome-wide meta-analysis of the association between NO_2 exposure during pregnancy and newborn cord blood DNA methylation (N=1,508 newborns from MeDALL, Generation R, CHS and MoBa cohorts).

Chr	Position (build 37)	CpG	Mapped gene	Gene group	Coef	SE	P-value	Direction
2	1482597	cg01385533	*TPO*	S_Shore	-0.003	8.00E-04	**0.0004**	-?--
2	1544120	cg19407717	*TPO*	N_Shore	-0.002	8.00E-04	**0.004**	----
2	1479523	cg13703866	*TPO*	N_Shore	-0.001	3.00E-04	**0.005**	----
2	1416855	cg08946720	*TPO*	OpenSea	-0.002	8.00E-04	**0.01**	--?+
2	1518383	cg24215279	*TPO*	S_Shelf	0.001	2.00E-04	**0.01**	+-+-
2	1488516	cg02892893	*TPO*	OpenSea	-0.001	5.00E-04	**0.02**	--?-
2	1425205	cg06972972	*TPO*	OpenSea	-0.001	4.00E-04	**0.02**	----
2	1478219	cg25013910	*TPO*	N_Shelf	-0.004	1.90E-03	**0.02**	--?-
2	1493713	cg06173919	*TPO*	Island	-0.002	9.00E-04	**0.04**	--?+
2	1482385	cg26140366	*TPO*	S_Shore	-0.002	9.00E-04	0.05	---+
2	1417153	cg12680131	*TPO*	OpenSea	-0.002	1.40E-03	0.09	----
2	1544321	cg04770020	*TPO*	Island	0.000	2.00E-04	0.11	---+
2	1494063	cg01681351	*TPO*	Island	-0.003	1.70E-03	0.11	??--
2	1500117	cg05678658	*TPO*	OpenSea	-0.002	1.20E-03	0.11	---+
2	1426181	cg03347837	*TPO*	OpenSea	-0.001	7.00E-04	0.12	-+-+
2	1493387	cg00626390	*TPO*	N_Shore	-0.001	6.00E-04	0.13	--?+
2	1482838	cg12666976	*TPO*	S_Shore	-0.002	1.50E-03	0.13	-++-
2	1497815	cg13414059	*TPO*	S_Shelf	-0.002	1.00E-03	0.16	-+--
2	1417109	cg16016036	*TPO*	OpenSea	-0.001	7.00E-04	0.16	-+--
2	1516247	cg07713008	*TPO*	Island	-0.001	1.00E-03	0.16	----
2	1494263	cg23596425	*TPO*	S_Shore	-0.003	1.90E-03	0.17	--++
2	1416889	cg00040862	*TPO*	OpenSea	0.001	8.00E-04	0.20	-++-
2	1452665	cg19368625	*TPO*	OpenSea	-0.001	1.10E-03	0.21	--++
2	1426094	cg14108581	*TPO*	OpenSea	-0.001	1.00E-03	0.21	--?-
2	1417431	cg10370591	*TPO*	OpenSea	0.001	6.00E-04	0.21	++++
2	1482915	cg23985797	*TPO*	S_Shore	0.001	9.00E-04	0.21	+-++
2	1507739	cg10672136	*TPO*	OpenSea	-0.001	1.20E-03	0.24	+--+
2	1488252	cg09337427	*TPO*	OpenSea	0.001	7.00E-04	0.24	+-+-
2	1544370	cg18995558	*TPO*	Island	-0.001	6.00E-04	0.25	----
2	1426068	cg10531568	*TPO*	OpenSea	-0.001	7.00E-04	0.26	-++-
2	1507694	cg15375772	*TPO*	OpenSea	-0.002	1.70E-03	0.27	+--+
2	1498138	cg03545077	*TPO*	S_Shelf	0.001	1.30E-03	0.27	++?+
2	1498065	cg18019372	*TPO*	S_Shelf	-0.002	1.60E-03	0.27	---+
2	1418073	cg23136645	*TPO*	OpenSea	0.000	4.00E-04	0.29	-++-
2	1418028	cg09757588	*TPO*	OpenSea	0.000	4.00E-04	0.31	+---
2	1425319	cg02963613	*TPO*	OpenSea	-0.001	7.00E-04	0.33	--+-
2	1487936	cg19905414	*TPO*	OpenSea	-0.001	8.00E-04	0.34	--?-
2	1484593	cg08025464	*TPO*	S_Shelf	-0.003	2.90E-03	0.35	-+--
2	1544474	cg06697522	*TPO*	Island	-0.001	7.00E-04	0.39	+---
2	1544076	cg17518079	*TPO*	N_Shore	0.001	7.00E-04	0.39	++-+

2	1426328	cg14691886	*TPO*	OpenSea	-0.001	6.00E-04	0.40	-+?-
2	1482963	cg20957776	*TPO*	S_Shore	-0.001	1.30E-03	0.40	--++
2	1488175	cg17299636	*TPO*	OpenSea	-0.001	1.00E-03	0.43	--?-
2	1479763	cg02332115	*TPO*	N_Shore	-0.001	1.10E-03	0.44	----
2	1500255	cg21759048	*TPO*	OpenSea	-0.001	8.00E-04	0.44	--+-
2	1461560	cg15301057	*TPO*	OpenSea	-0.001	8.00E-04	0.44	-+?-
2	1543221	cg05090359	*TPO*	N_Shore	0.001	6.00E-04	0.45	+++-
2	1484125	cg21856680	*TPO*	S_Shelf	0.001	6.00E-04	0.46	+-+-
2	1513998	cg03133821	*TPO*	N_Shelf	0.000	6.00E-04	0.46	-+--
2	1543327	cg04319651	*TPO*	N_Shore	0.000	6.00E-04	0.47	---+
2	1452260	cg16407924	*TPO*	OpenSea	-0.001	1.90E-03	0.48	+-?-
2	1479810	cg01112527	*TPO*	N_Shore	-0.001	7.00E-04	0.48	----
2	1517047	cg14926103	*TPO*	S_Shore	0.001	7.00E-04	0.50	+--+
2	1494681	cg14011419	*TPO*	S_Shore	0.001	1.10E-03	0.52	++?-
2	1500368	cg17494408	*TPO*	OpenSea	0.000	7.00E-04	0.54	-++-
2	1489909	cg25250661	*TPO*	N_Shelf	-0.001	1.30E-03	0.56	+---
2	1417164	cg06500727	*TPO*	OpenSea	-0.001	9.00E-04	0.57	+--+
2	1516240	cg24798914	*TPO*	Island	0.000	4.00E-04	0.58	+---
2	1497906	cg02924495	*TPO*	S_Shelf	0.000	7.00E-04	0.59	++?+
2	1544352	cg06574769	*TPO*	Island	0.000	7.00E-04	0.60	--++
2	1497868	cg26541429	*TPO*	S_Shelf	0.000	7.00E-04	0.61	--+-
2	1488565	cg04658693	*TPO*	OpenSea	0.000	7.00E-04	0.61	-++-
2	1418294	cg15977002	*TPO*	OpenSea	0.000	7.00E-04	0.68	+---
2	1426845	cg07088935	*TPO*	OpenSea	0.000	1.00E-03	0.68	++--
2	1542097	cg01028140	*TPO*	N_Shelf	0.000	9.00E-04	0.69	--++
2	1507832	cg23239637	*TPO*	OpenSea	0.000	2.00E-04	0.71	--+-
2	1543996	cg11275685	*TPO*	N_Shore	0.000	6.00E-04	0.72	--++
2	1481097	cg14601038	*TPO*	Island	-0.001	2.10E-03	0.72	--++
2	1480789	cg07932899	*TPO*	Island	-0.001	1.80E-03	0.73	-+++
2	1487895	cg19585646	*TPO*	OpenSea	0.000	1.00E-03	0.73	++?-
2	1488638	cg14935163	*TPO*	OpenSea	0.000	3.00E-04	0.76	++--
2	1425035	cg14478255	*TPO*	OpenSea	0.000	7.00E-04	0.77	-+-+
2	1425560	cg26277787	*TPO*	OpenSea	0.000	7.00E-04	0.80	++--
2	1481492	cg08537127	*TPO*	Island	0.001	5.50E-03	0.81	?-?+
2	1417248	cg07083862	*TPO*	OpenSea	0.000	8.00E-04	0.86	+-+-
2	1543546	cg21913853	*TPO*	N_Shore	0.000	5.00E-04	0.87	++--
2	1452367	cg26605809	*TPO*	OpenSea	0.000	1.30E-03	0.88	-+++
2	1482185	cg21997141	*TPO*	S_Shore	0.000	8.00E-04	0.89	--+-
2	1426301	cg05544715	*TPO*	OpenSea	0.000	3.00E-04	0.92	-+--
2	1426674	cg24216893	*TPO*	OpenSea	0.000	8.00E-04	0.92	+---
2	1516061	cg01312658	*TPO*	N_Shore	0.000	5.00E-04	0.96	--+-
2	1480944	cg14793137	*TPO*	Island	0.000	2.80E-03	0.97	-+++
2	1415911	cg25349955	*TPO*	OpenSea	0.000	6.00E-04	0.97	+--+
2	1425620	cg03795587	*TPO*	OpenSea	0.000	6.00E-04	0.98	++--
2	1516352	cg13661012	*TPO*	Island	0.000	4.00E-04	0.99	+-+-
2	1488314	cg04152326	*TPO*	OpenSea	0.000	5.00E-04	1.00	++--

Shown are all CpGs within *TPO* gene ordered by p-value, with exception of cg01957222 only available in MoBa cohort. All results presented per 10 μg/m^3 increase in prenatal NO_2 exposure.

Column headers: CHR: chromosome; Mapped Gene: UCSC annotated gene; Gene Group: UCSC gene region feature category; Coef: regression coefficient; Direction: Direction of effect across cohorts included in the statistical model (MeDALL, Generation R, CHS and MoBa): NO_2 exposure during pregnancy associated with increased (+) or decreased (-) methylation, or missing (?) result.

Table S9. Look-up in older children for top CpGs in the *CAT* and *TPO* genes for prenatal NO_2 exposure in relation to methylation in meta-analysis of newborns (n=1,508).

Meta-analysis of cord blood results (MeDALL + Generation R + CHS + MoBa)									Replication in 4-year-old children of the MeDALL cohorts						Replication in 8-year-old children of the MeDALL + EpiGene cohorts (meta-analysis)					
NO_2 pregnancy → Methylation at birth (n=1,508)									NO_2 at pregnancy→ Methylation at 4 yrs (n=733)			NO_2 at 4 yrs → Methylation at 4 yrs (n=689)			NO_2 at pregnancy → Methylation at 8 yrs (n=786)			NO_2 at 8 yrs → Methylation at 8 yrs (n=829)		
**Chr**	**Position (build 37)**	**CpG**	**Mapped gene**	**Gene group**	**Coef**	**SE**	**P-value**	**Direction**	**Coef**	**SE**	**P-value**	**Coef**	**SE**	**P-value**	**Coef**	**SE**	**P-value**	**Coef**	**SE**	**P-value**
11	34460856	cg03728580	*CAT* [FDR]	Body	0.003	7.00E-04	0.00001	++++	0.0000	9.62E-04	1.00	0.000	1.23E-03	0.85	0.0000	1.00E-03	0.97	0.0006	1.60E-03	0.68
11	34461028	cg17034036	*CAT* [FDR]	Body	0.002	6.00E-04	0.0001	++++	0.0003	7.78E-04	0.72	-0.0001	8.47E-04	0.93	0.0002	1.00E-03	0.82	-0.0003	1.20E-03	0.80
2	1482597	cg01385533	*TPO* [FDR]	Body	-0.003	8.00E-04	0.0004	-?--	-0.0006	7.31E-04	0.40	-0.002	7.87E-04	**0.04**	-0.0018	8.00E-04	**0.04**	-0.0016	1.30E-03	0.22

Shown are top CpGs within the *CAT* and *TPO* genes from the discovery meta-analysis ordered by p-value; Estimates are presented per 10 µg/m^3 increase in NO_2 exposure.

Column headers: CHR: chromosome; Position: Chromosomal position based on NCBI human reference genome assembly Build 37. Mapped Gene: UCSC annotated gene; Gene Group: UCSC gene region feature category; regression coefficient; SE: standard error for regression coefficient; Direction: Direction of effect across cohorts included in the statistical model (MeDALL, Generation R, CHS and MoBa): NO_2 exposure associated with increased (+) or decreased (-) methylation, or missing (?) result.

Figure S3

A)

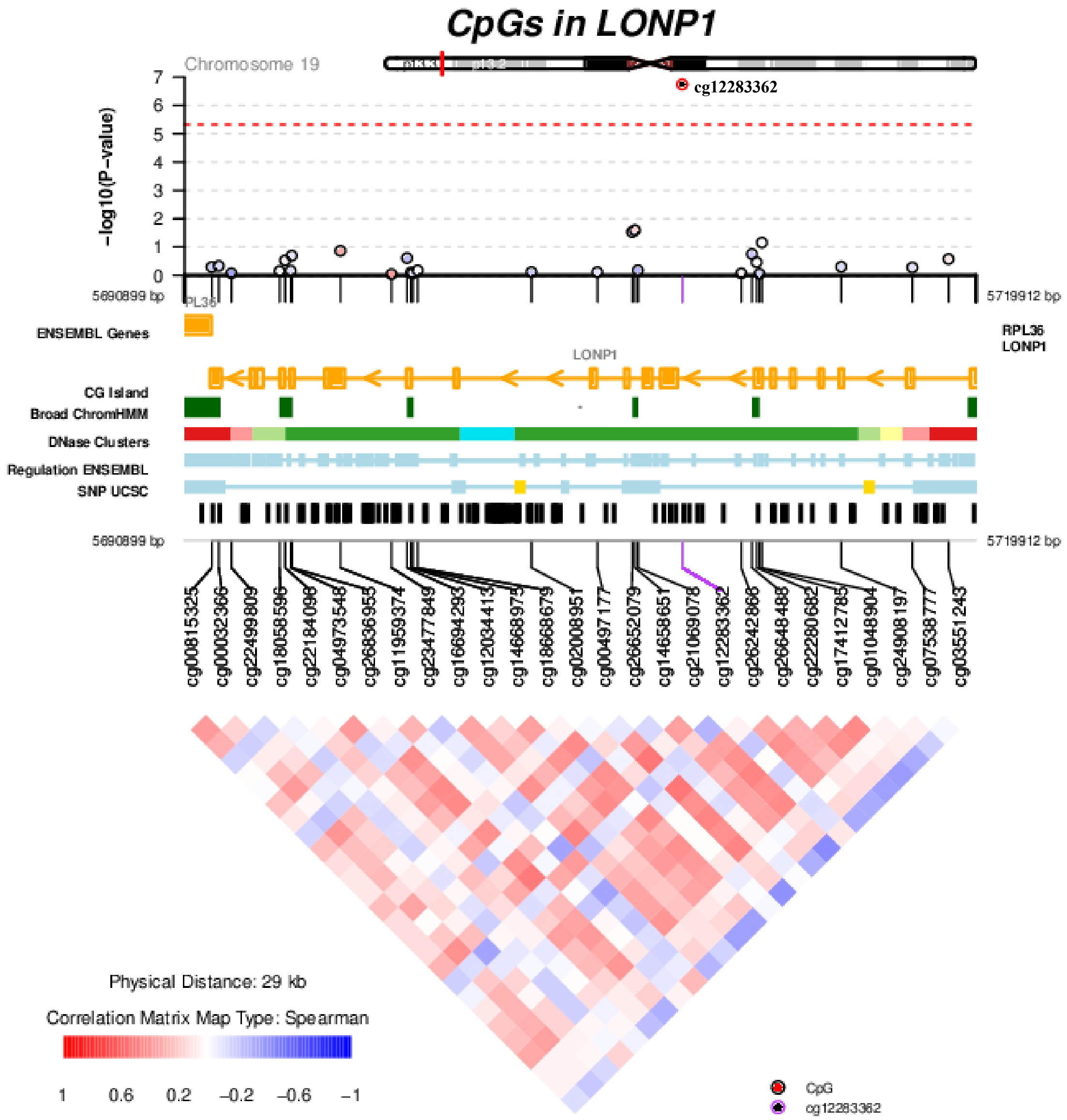

CpGs in LONP1
Chromosome 19
cg12283362
-log10(P-value)
5690899 bp
5719912 bp
ENSEMBL Genes
RPL36
LONP1
CG Island
Broad ChromHMM
DNase Clusters
Regulation ENSEMBL
SNP UCSC
cg00815325
cg00032366
cg22499809
cg18058596
cg22184096
cg04973548
cg26836955
cg11959374
cg23477849
cg16694293
cg12034413
cg14668975
cg18668679
cg02008951
cg00497177
cg26652079
cg14658651
cg21069078
cg12283362
cg26242866
cg26648488
cg22280682
cg17412785
cg01048904
cg24908197
cg07538777
cg03551243
Physical Distance: 29 kb
Correlation Matrix Map Type: Spearman
1 0.6 0.2 -0.2 -0.6 -1
CpG
cg12283362

B)

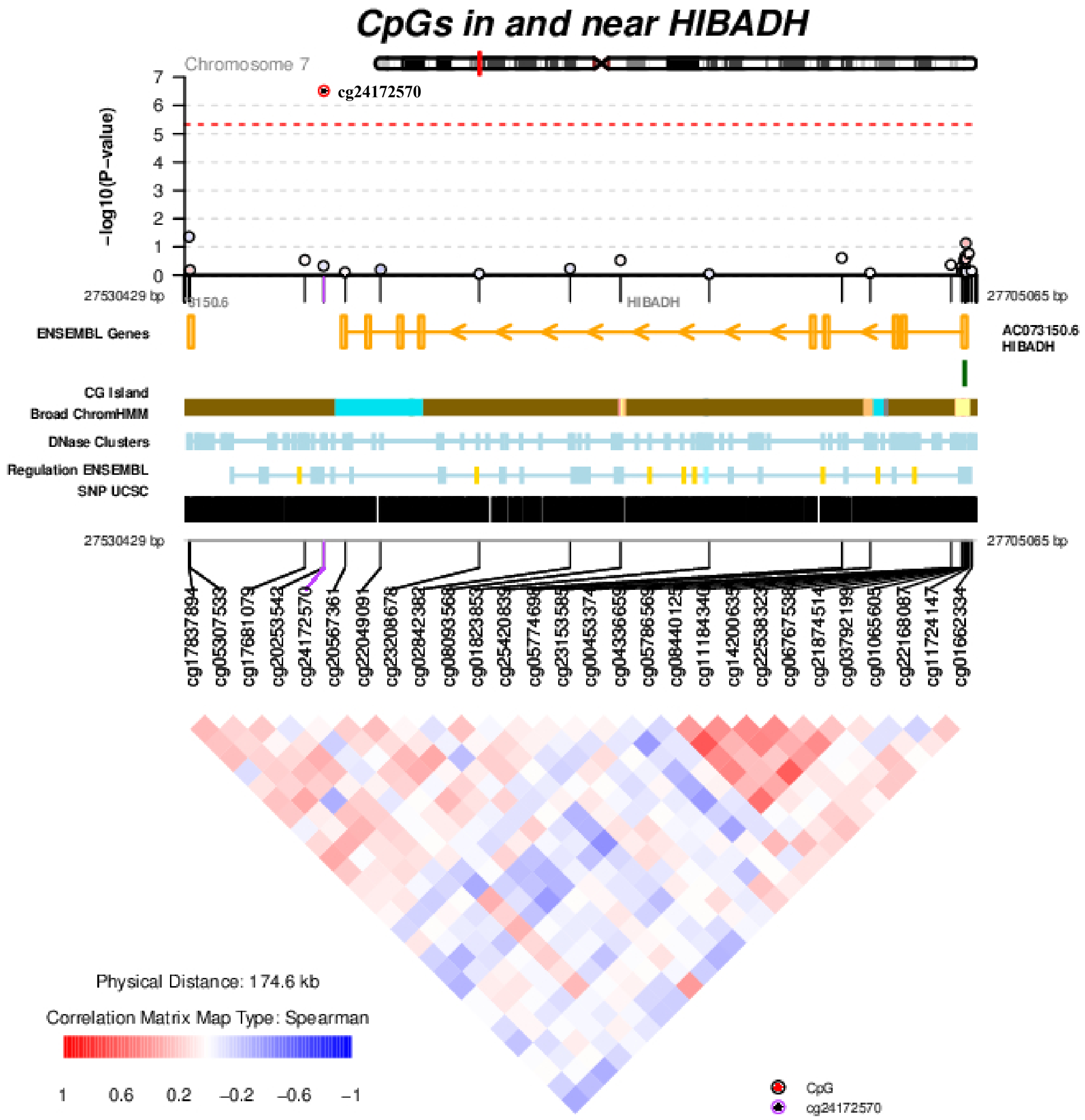

CpGs in and near HIBADH
Chromosome 7
cg24172570
−log10(P−value)
27530429 bp
27705065 bp
HIBADH
ENSEMBL Genes
AC073150.6
HIBADH
CG Island
Broad ChromHMM
DNase Clusters
Regulation ENSEMBL
SNP UCSC
Physical Distance: 174.6 kb
Correlation Matrix Map Type: Spearman
1 0.6 0.2 −0.2 −0.6 −1
CpG
cg24172570

C)

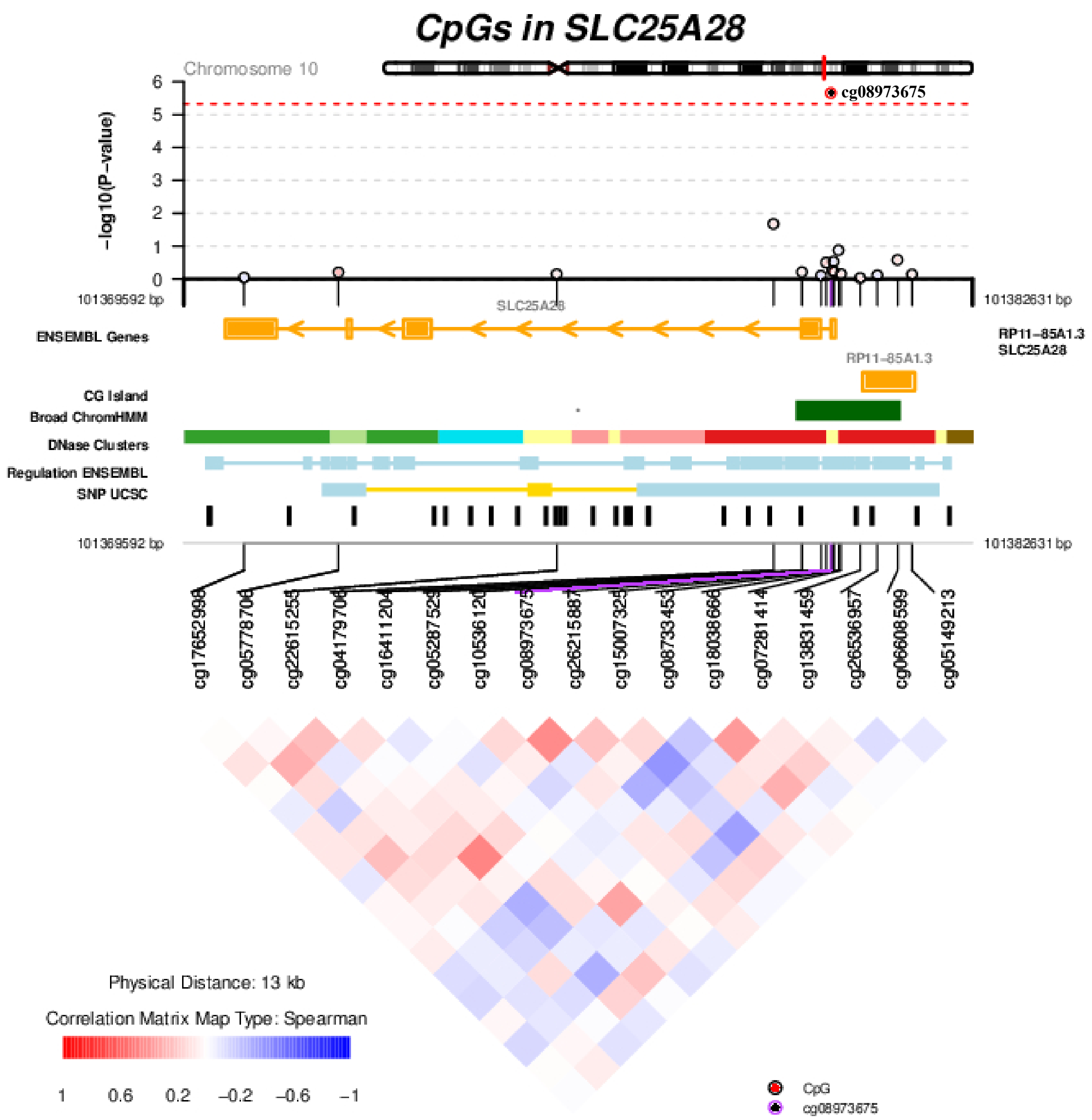
CpGs in SLC25A28
Chromosome 10
cg08973675
-log10(P-value)
101369592 bp
101382631 bp
SLC25A28
ENSEMBL Genes
RP11-85A1.3
SLC25A28
RP11-85A1.3
CG Island
Broad ChromHMM
DNase Clusters
Regulation ENSEMBL
SNP UCSC
cg17652998
cg05778706
cg22615255
cg04179706
cg16411204
cg05287525
cg10536120
cg08973675
cg26215887
cg15007325
cg08733453
cg18038666
cg07281414
cg13831459
cg26536957
cg06608599
cg05149213
Physical Distance: 13 kb
Correlation Matrix Map Type: Spearman
1 0.6 0.2 -0.2 -0.6 -1
CpG
cg08973675

D)

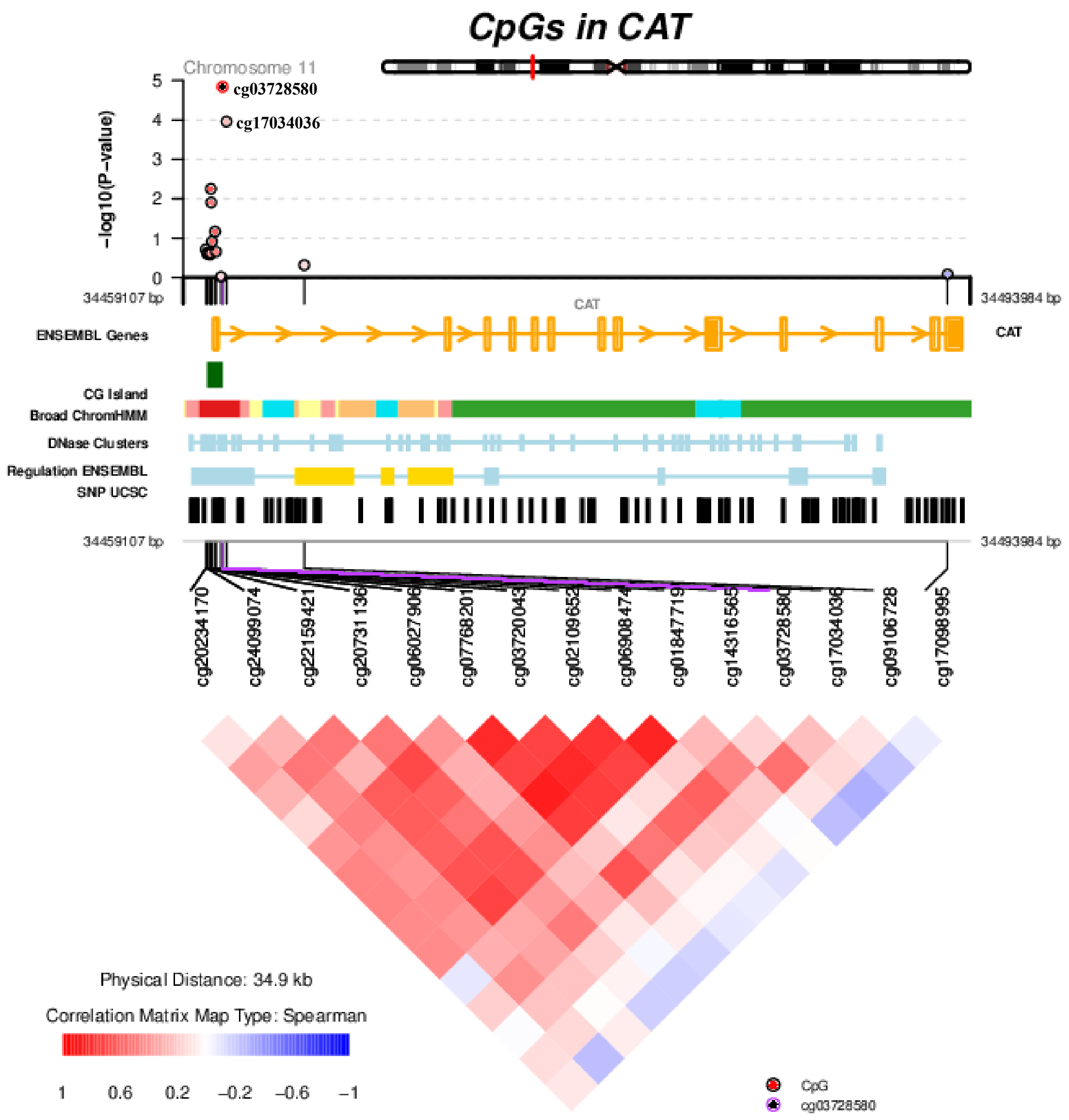

CpGs in CAT
Chromosome 11
cg03728580
cg17034036
-log10(P-value)
5
4
3
2
1
0
34459107 bp
34493984 bp
CAT
ENSEMBL Genes
CAT
CG Island
Broad ChromHMM
DNase Clusters
Regulation ENSEMBL
SNP UCSC
34459107 bp
34493984 bp
cg20234170
cg24099074
cg22159421
cg20731136
cg06027906
cg07768201
cg03720043
cg02109652
cg06908474
cg01847719
cg14316565
cg03728580
cg17034036
cg09106728
cg17098995
Physical Distance: 34.9 kb
Correlation Matrix Map Type: Spearman
1
0.6
0.2
-0.2
-0.6
-1
CpG
cg03728580

E)

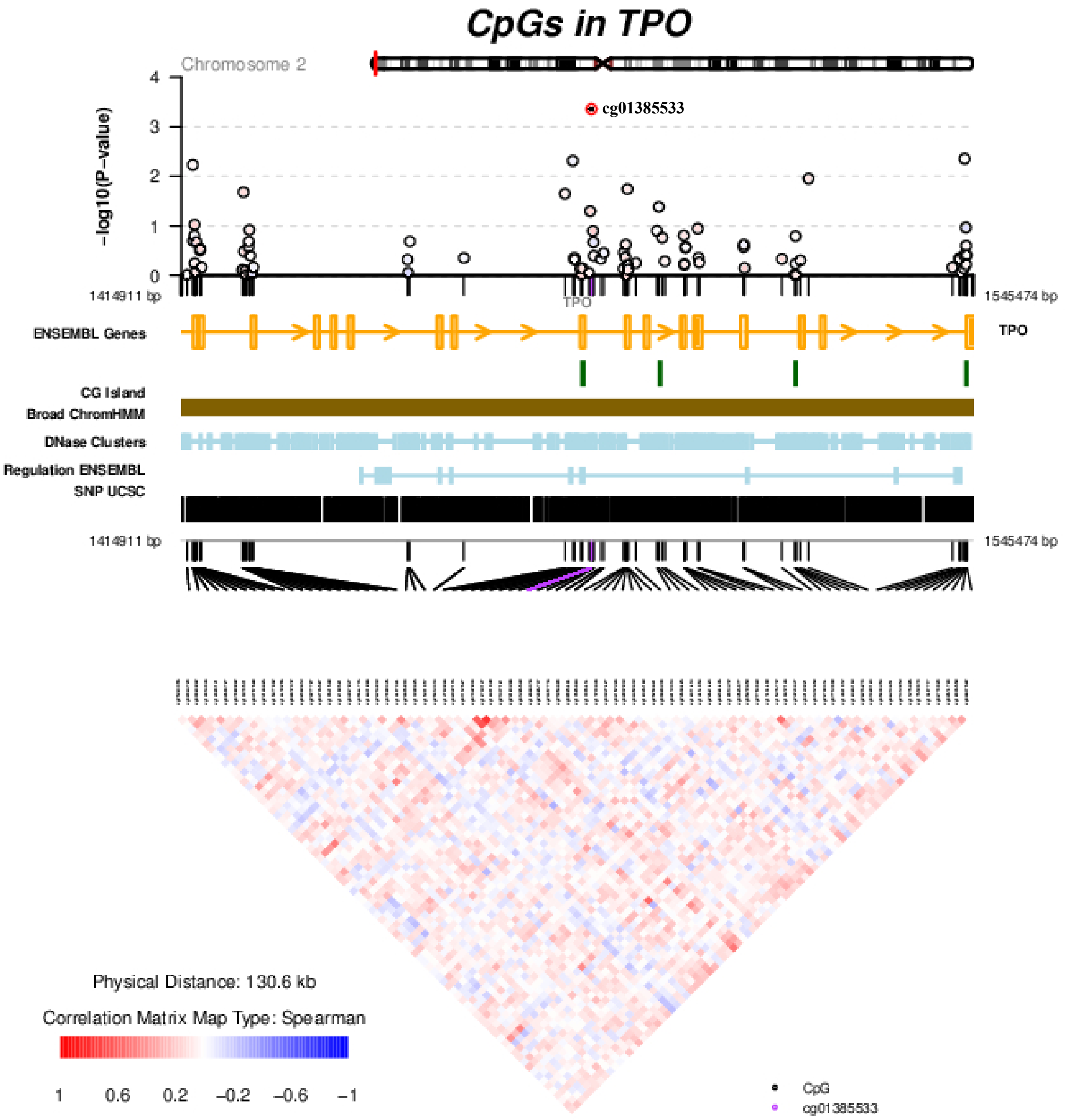

Figure S3. Regional plots and co-methylation patterns for the genes containing FDR-significant EWAS CpG sites (circles): A) *LONP1* (cg12283362), B) *HIBADH* (cg24172570), C) *SLC25A28* (cg08973675), as well as D) *CAT* (cg03728580 and cg17034036) and E) *TPO* (cg01385533). -log10(p values) from the meta-analysis, CpGs indicated by dots, color coded based on pairwise correlation with neighboring CpGs. The lower panel demonstrate pairwise correlation matrix across the displayed CpGs.

Acknowledgements

INMA: We would like thank all the families and children for their participation in the INMA study. The authors are grateful to Silvia Fochs, NuriaPey, and Muriel Ferrer, for their assistance in contacting the families and administering the questionnaires. A full roster of the INMA project investigators can be found at http://www.proyectoinma.org/presentacion-inma/listado-investigadores/en_listado-investigadores.html. DNA extractions were performed at the Spanish National Genotyping Centre (CEGEN-Barcelona).

EDEN: We are indebted to the midwife research assistants (L. Douhaud, S. Bedel, B. Lortholary, S. Gabriel, M. Rogeon, and M. Malinbaum) for data collection and to P. Lavoine for checking, coding, and data entry.

BAMSE: We would like thank all the families for their participation in the BAMSE study. In addition, we would like to thank Eva Hallner, Sara Nilsson and André Lauber at the BAMSE secretary for invaluable support, as well as Mutation Analysis Facility (MAF) at Karolinska Institutet for genome-wide methylation analysis, and Ingrid Delin for excellent technical assistance. The computations were performed on resources provided by SNIC through Uppsala Multidisciplinary Center for Advanced Computational Science (UPPMAX) under Project b2014110.

The Generation R Study is conducted by the Erasmus Medical Center (MC) in close collaboration with the School of Law and Faculty of Social Sciences of the Erasmus University Rotterdam, the Municipal Health Service Rotterdam area, Rotterdam, the Rotterdam Homecare Foundation, Rotterdam and the Stichting Trombosedienst & Artsenlaboratorium Rijnmond (STAR-MDC), Rotterdam. We gratefully acknowledge the contribution of children and parents, general practitioners, hospitals, midwives and pharmacies in Rotterdam. The study protocol was approved by the Medical Ethical Committee of the Erasmus MC, University Medical Centre, Rotterdam, the Netherlands. The generation and management of the Illumina 450K methylation array data (EWAS data) for the

Generation R Study was executed by the Human Genotyping Facility of the Genetic Laboratory of the Department of Internal Medicine, Erasmus MC, Erasmus University Medical Center, Rotterdam, the Netherlands. We thank Mr. Michael Verbiest, Ms. Mila Jhamai, Ms. Sarah Higgins, Mr. Marijn Verkerk and Dr. Lisette Stolk for their help in creating the EWAS database.

MoBa, we are grateful to all families participating in the Norwegian Mother and Child Cohort Study.

Funding

The methylation study of PIAMA, BAMSE, EDEN and INMA birth cohorts was funded by MEDALL, a collaborative project supported by the European Union under the Health Cooperation Work Programme of the 7th Framework programme (grant agreement number 261357).

INMA study was funded by grants from Instituto de Salud Carlos III (Red INMA G03/176, CB06/02/0041; PI041436; PI081151 incl. FEDER funds ; MS13/00054), Generalitat de Catalunya-CIRIT 1999SGR 00241, and EU Commission (261357 and 211250).

The PIAMA study was funded by grants from the Dutch Asthma Foundation (grant 3.4.01.26, 3.2.06.022, 3.4.09.081 and 3.2.10.085CO), the ZON-MW Netherlands Organization for Health Research and Development (grant 912-03-031), the Stichting Astmabestrijding and the Ministry of the Environment.

The EDEN cohort is funded by FRM, Inserm, IReSP, Nestlé, French Ministry of Health, ANR, Univ. Paris-Sud, InVS, ANSES and MGEN.

BAMSE was supported by The Swedish Research Council, The Swedish Heart-Lung Foundation, Freemason Child House Foundation in Stockholm, MeDALL (Mechanisms of the Development of ALLergy) a collaborative project conducted within the European Union

(grant agreement No. 261357), Centre for Allergy Research, Stockholm County Council (ALF), Swedish foundation for strategic research (SSF) (RBc08-0027), the Strategic Research Programme (SFO) in Epidemiology at Karolinska Institutet, The Swedish Research Council Formas and the Swedish Environment Protection Agency.

The Generation R Study is made possible by financial support from the Erasmus Medical Center (MC), Rotterdam, the Erasmus University Rotterdam and the Netherlands Organization for Health Research and Development. V.W.V.J. received an additional grant from the Netherlands Organization for Health Research and Development (VIDI 016.136.361) and a Consolidator Grant from the European Research Council (ERC-2014-CoG-64916). L.D. received an additional grant from the Lung Foundation Netherlands (no 3.2.12.089; 2012). J.F.F. has received funding from the European Union's Horizon 2020 research and innovation programme under grant agreement No 633595 (DynaHEALTH). The EWAS data was funded by a grant to V.W.V.J. from the Netherlands Genomics Initiative (NGI)/Netherlands Organisation for Scientific Research (NWO) Netherlands Consortium for Healthy Aging (NCHA; project nr. 050-060-810), and by funds from the Genetic Laboratory of the Department of Internal Medicine, Erasmus Medical Center. The researchers are independent from the funders. The study sponsors had no role in the study design, data collection, data analysis, interpretation of data, and preparation, review or approval of the manuscript.

The CHS was supported by the following NIH grants: 5K01ES017801, 1R01ES022216, 5P30ES007048, R01ES014447, P01ES009581, R826708-01 and RD831861-01. We would like to express our sincere gratitude to Martin Kharrazi, Steve Graham and Robin Cooley at the California Biobank Program and Genetic Disease Screening Program within the California Department of Public Health for their assistance and advice regarding newborn bloodspots.

For MoBa this research was supported [in part] by the Intramural Research Program of the NIH, National Institute of Environmental Health Sciences (Z01-ES-49019). The Norwegian Mother and Child Cohort Study is supported by the Norwegian Ministry of Health and the Ministry of Education and Research, NIH/NIEHS (contract no. N01-ES-75558), NIH/NINDS (grant no.1 UO1 NS 047537-01) and the Norwegian Research Council/FUGE (grant no. 151918/S10), and the present study by the Norwegian Research Council/Human Biobanks and Health (grant number 221097).

References

Aryee MJ, Jaffe AE, Corrada-Bravo H, et al. 2014. Minfi: A flexible and comprehensive bioconductor package for the analysis of infinium DNA methylation microarrays. Bioinformatics 30:1363-1369.

Beelen R, Hoek G, Vienneau D, et al. 2013. Development of no2 and nox land use regression models for estimating air pollution exposure in 36 study areas in europe - the escape project. Atmos Environ 72:10-23.

Bolstad BM, Irizarry RA, Astrand M, et al. 2003. A comparison of normalization methods for high density oligonucleotide array data based on variance and bias. Bioinformatics 19:185-193.

Bousquet J, Anto J, Auffray C, et al. 2011. Medall (mechanisms of the development of allergy): An integrated approach from phenotypes to systems medicine. Allergy 66:596-604.

Chen YA, Lemire M, Choufani S, et al. 2013. Discovery of cross-reactive probes and polymorphic cpgs in the illumina infinium humanmethylation450 microarray. Epigenetics 8:203-209.

Guxens M, Ballester F, Espada M, et al. 2012. Cohort profile: The inma--infancia y medio ambiente--(environment and childhood) project. Int J Epidemiol 41:930-940.

Hannam K, McNamee R, De Vocht F, et al. 2013. A comparison of population air pollution exposure estimation techniques with personal exposure estimates in a pregnant cohort. Environ Sci Process Impacts 15:1562-1572.

Heude B, Forhan A, Slama R, et al. 2015. Cohort profile: The eden mother-child cohort on the prenatal and early postnatal determinants of child health and development. Int J Epidemiol.

Jaddoe VW, van Duijn CM, Franco OH, et al. 2012. The generation r study: Design and cohort update 2012. Eur J Epidemiol 27:739-756.

Joubert B, Felix J, Yousefi P, et al. 2016. DNA methylation in newborns and maternal smoking in pregnancy: Genome-wide consortium meta-analysis. Am J Hum Genet 98(4):680-696.

Kruithof CJ, Kooijman MN, van Duijn CM, et al. 2014. The generation r study: Biobank update 2015. Eur J Epidemiol 29:911-927.

Magnus P, Irgens LM, Haug K, et al. 2006. Cohort profile: The norwegian mother and child cohort study (moba). Int J Epidemiol 35:1146-1150.

McConnell R, Berhane K, Yao L, et al. 2006. Traffic, susceptibility, and childhood asthma. Environ Health Perspect 114:766-772.

Noushmehr H, Weisenberger DJ, Diefes K, et al. 2010. Identification of a cpg island methylator phenotype that defines a distinct subgroup of glioma. Cancer Cell 17:510-522.

Pedersen M, Giorgis-Allemand L, Bernard C, et al. 2013. Ambient air pollution and low birthweight: A european cohort study (escape). The lancet Respiratory medicine 1:695-704.

Peters JM, Avol E, Gauderman WJ, et al. 1999a. A study of twelve southern california communities with differing levels and types of air pollution. Ii. Effects on pulmonary function. Am J Respir Crit Care Med 159:768-775.

Peters JM, Avol E, Navidi W, et al. 1999b. A study of twelve southern california communities with differing levels and types of air pollution. I. Prevalence of respiratory morbidity. Am J Respir Crit Care Med 159:760-767.

Pidsley R, CC YW, Volta M, et al. 2013. A data-driven approach to preprocessing illumina 450k methylation array data. BMC Genomics 14:293.

Rivera-Gonzalez LO, Zhang Z, Sanchez BN, et al. 2015. An assessment of air pollutant exposure methods in mexico city, mexico. J Air Waste Manag Assoc 65:581-591.

Ronningen KS, Paltiel L, Meltzer HM, et al. 2006. The biobank of the norwegian mother and child cohort study: A resource for the next 100 years. Eur J Epidemiol 21:619-625.

Thacher JD, Gruzieva O, Pershagen G, et al. 2016. Parental smoking and development of allergic sensitization from birth to adolescence. Allergy 71:239-248.

Touleimat N, Tost J. 2012. Complete pipeline for infinium((r)) human methylation 450k beadchip data processing using subset quantile normalization for accurate DNA methylation estimation. Epigenomics 4:325-341.

Triche TJ, Jr., Weisenberger DJ, Van Den Berg D, et al. 2013. Low-level processing of illumina infinium DNA methylation beadarrays. Nucleic Acids Res 41:e90.

Van den Hooven EH, Pierik FH, Van Ratingen SW, et al. 2012. Air pollution exposure estimation using dispersion modelling and continuous monitoring data in a prospective birth cohort study in the netherlands. Environ Health-Glob 11:9.

Wickman M, Kull I, Pershagen G, et al. 2002. The bamse project: Presentation of a prospective longitudinal birth cohort study. Pediatr Allergy Immunol 13 Suppl 15:11-13.
